# Sdfconf: A Novel, Flexible, and Robust Molecular Data Management Tool

**DOI:** 10.1021/acs.jcim.1c01051

**Published:** 2021-12-21

**Authors:** Sakari T. Lätti, Sanna Niinivehmas, Olli T. Pentikäinen

**Affiliations:** † Institute of Biomedicine, Faculty of Medicine, University of Turku, FI-20520 Turku, Finland; ‡ InFLAMES Research Flagship Center, University of Turku, FI-20520 Turku, Finland; § Aurlide ltd, FI-21420 Lieto, Finland

## Abstract

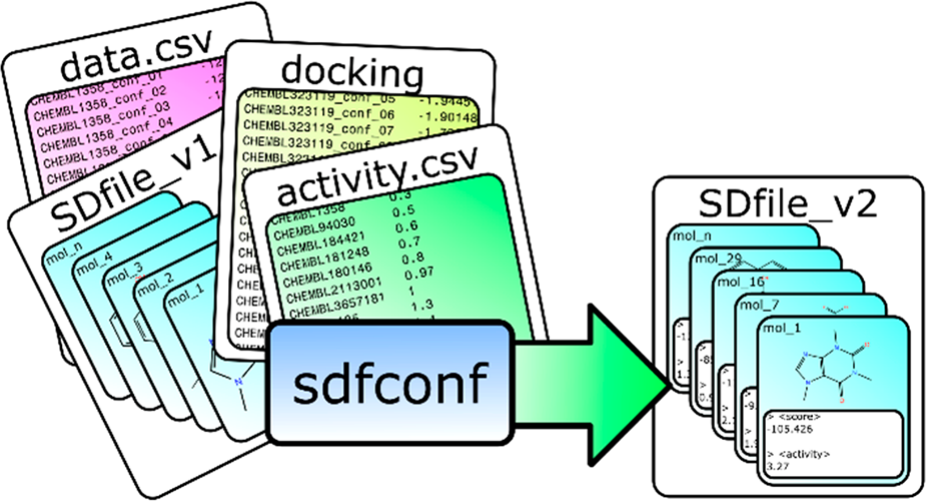

Projects in chemo- and bioinformatics often consist of scattered data in various types and are difficult to access in a meaningful way for efficient data analysis. Data is usually too diverse to be even manipulated effectively. Sdfconf is data manipulation and analysis software to address this problem in a logical and robust manner. Other software commonly used for such tasks are either not designed with molecular and/or conformational data in mind or provide only a narrow set of tasks to be accomplished. Furthermore, many tools are only available within commercial software packages. Sdfconf is a flexible, robust, and free-of-charge tool for linking data from various sources for meaningful and efficient manipulation and analysis of molecule data sets. Sdfconf packages molecular structures and metadata into a complete ensemble, from which one can access both the whole data set and individual molecules and/or conformations. In this software note, we offer some practical examples of the utilization of sdfconf.

## Introduction

Types and volumes of computational data have exploded when both computation capacity and utilization of computational methods have increased. This has led to substantial data management and analysis challenges, especially in cases when there is no mature custom workflow. Many research institutions, funding agencies, or individual projects have set official guidelines for managing and handling scientific data. In chemo- and bioinformatics fields, projects are often burdened with large amounts of data from various sources. This data is typically large and complex enough to be challenging to handle and utilize effectively but still too small to construct custom databases and workflows for it.

First, molecular structural data is usually processed in multiple steps, starting from initial structures and ending in processed conformations. Second, there are data related to each compound, like experimental values (e.g., activity) and calculated physicochemical properties (e.g., molecular mass). Third, there is data associated with molecular conformations (e.g., scores from docking software). Finally, in some projects, there are atomic data both on the molecular and conformational levels. Usually, it is very tedious to link and utilize data acquired in different levels and steps, and thus, simple and practical tools for data management and analysis are needed.

**Figure 1:**
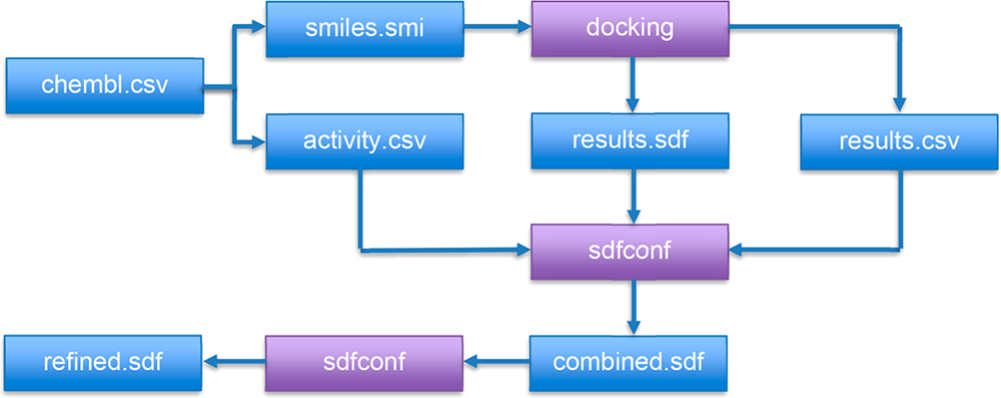
Simple example workflow for molecular docking and result from refinement and/or analysis. Molecules with activity data are docked, and then activity, docked structures, and docking scores are combined into one SDfile. Then conformations may be chosen, and results may be refined and analyzed.

Sdfconf aims to provide an easy-to-use command-line tool for linking and managing data from various sources for meaningful and efficient manipulation of molecule data sets (Figure 1). The main strength of sdfconf is its ability to access and utilize every bit of information the user is interested in. Sdfconf is aimed to be a general rather than a special tool. For this purpose, sdfconf features so-called metastaments utilized in most of its use cases. A metastatement is a single expression that refers to specific information that is applicable and meaningful for the whole data set of multiple molecules and conformations.

Sdfconf is constructed for handling SDfiles (.sdf) with molfiles of V2000 specification. It has methods for reading, importing, manipulation, analysis, exportation, and plotting of .sdf and .csv files. In certain tasks with external files, also .mol2 and .pdb file formats are supported. Sdfconf utilizes an extended notation of SDfile property data item, which is called metadata or meta for short in the context of sdfconf. The extended notation is compatible with the standard but will often be interpreted as text. Different structures and data types for metadata raise the need to use this data effectively. One of the most important features of sdfconf is its diverse way of referring to wanted metadata. With a metastatement, we can assign information not only for the whole molecule but its atoms too. Sdfconf also has a mechanism for distinguishing molecules as a set of groups, e.g., multiple molecules with the same name, each having multiple conformations. Therefore, one can handle molecular, conformational, and atomic data at the same time.

Typical problems that sdfconf can solve include:

1. Spatial requirements for docked molecules, including distances and overlaps
2. Combining conformational data from various sources
3. Complex renaming of molecules depending on conformation properties
4. Complex schemes for picking (and defining) molecule conformations, e.g., by spatial proximity
5. Revealing interesting (trending) points in protein–ligand complexes
6. Finding a correlation between molecular and conformational properties
7. Storing data related to molecules, molecule conformations, and atoms

## Implementation

**Figure 2:**
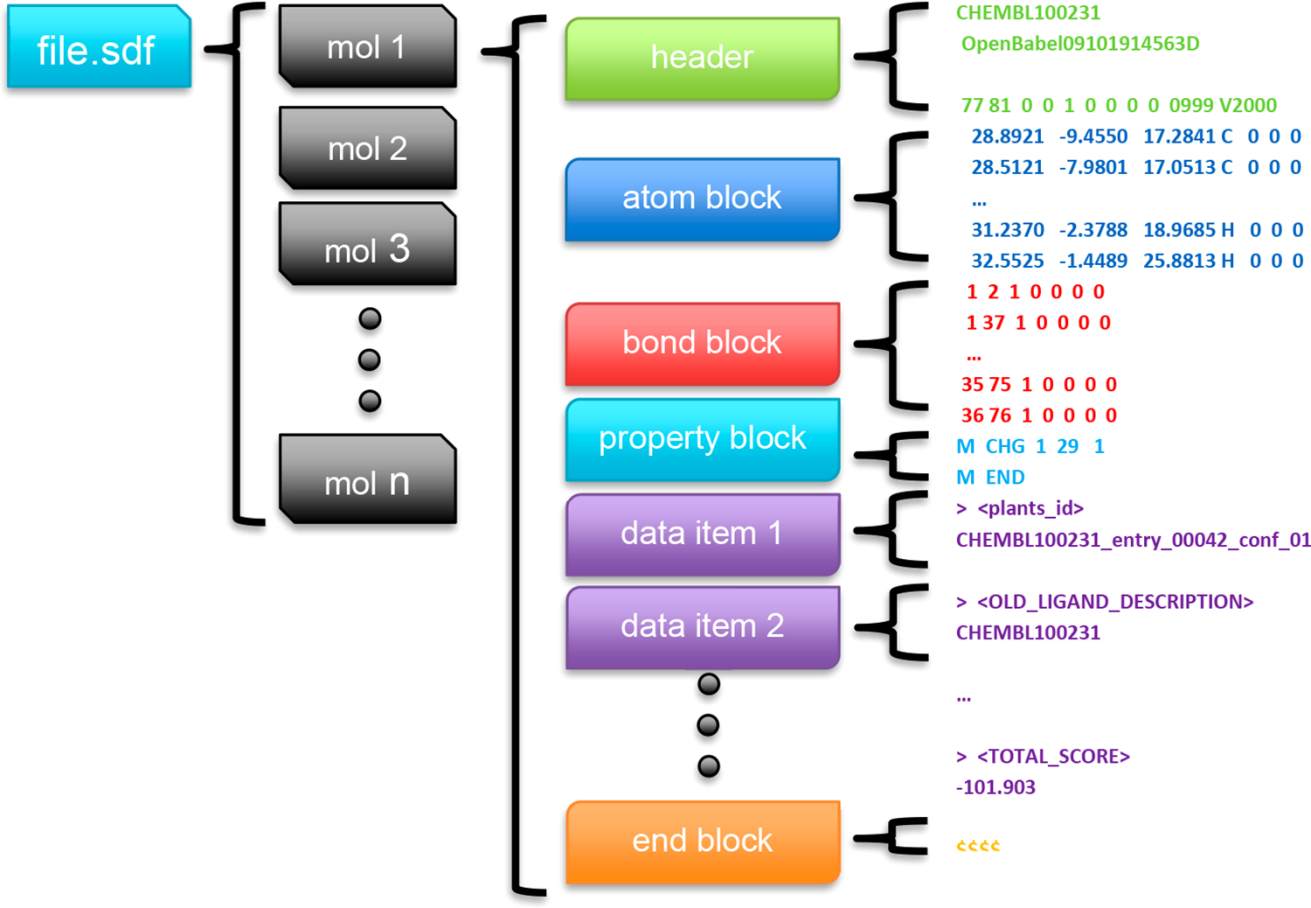
Structure of V2000 molfile. In its basic form, V2000-molfile can store header block (molecule name, two comment lines, and counts) and connection table (atom block, bond block, and property block). Molfile may be followed by a number of data items. In an SDfile, a single molfile with a number of data items ends with signal $$$$.

Sdfconf is written in python 2.7. It uses a few well-established libraries like NumPy and matplotlib for its core functions. It uses mainly SDfiles with V2000 specified molfiles^1^ (Figure 2), and many of its properties are implemented in such a way that data can be easily stored in them. Sdfconf uses the relatively free format of data items to store all data that cannot be stored elsewhere in an SDfile. Sdfconf also has some tools for usage with mol2-files. SDfile format specifies properties for molecules. Properties are, in general singular piece of data that has a name, like “mol_weight” and value like “174.05”. Usually, an SDfile includes a certain set of properties for all molecules in the file. In sdfconf, properties are called metadata.

The extended notation in sdfconf uses three types of data structure: single, list, and dictionary. Single and list are self-explanatory, but the dictionary includes a list of key and value pairs. For example, a key could be an atom number and the corresponding value distance between the specified atom and some point-of-interest (like a point inside the binding site of a protein).

Sdfconf can store, manipulate, and utilize molecule-, conformation-, and atom-wise data in a way referred to as metadata. All stored metadata has a name and a value. Metadata can never exist without a value, i.e., there is no metadata if there is no value. This is due to the format itself. The data value may be integer, floating-point, or string. Those may be stored as the aforementioned single value, list, or dictionary. Dictionaries can store arbitrary keys and values if imported correctly into sdfconf, but sdfconf itself uses them to store atom-wise data. In the latter case, meta is associated with certain conformation, and keys are atom numbers.

A metastatement can assign this data in a very flexible yet robust manner. The result of a metastatement is always metadata itself, although it does not have a name, and therefore it is not saved unless it is assigned into the file as a new data item. However, an unsaved metastatement may be referred to, as metadata would be. A statement can include an arbitrary amount of metadata and build-in sdfconf functions to create new metadata that can be further utilized in (another) metastatement. In other words, metastatements can be nested. The metanotation also supports three data types: integer, floating-point number, and string. Sdfconf tries to interpret data in this order.

Sdfconf may be used in virtually any system with python 2.7 and its common libraries. When installed, sdfconf is handled as a python package, so one can also call sdfconf as a python library in other python programs. Therefore, sdfconf can also be used with a python interactive interpreter to be used in an even more flexible manner.

## Results and Discussion

By default, sdfconf is a CLI (command-line interface) tool, and currently, there are no plans to change that. Functions similar to sdfconf may be achieved with other software featuring graphical user interfaces (e.g., datawarrior^2^), but one loses the benefit of repeatability that way. As the user learns to achieve desired functions with CLI, one has already made the task easily replicable via scripting.

At the core of sdfconf are metastatements, which enable the user to access data in an effective manner. Details of the notation are not discussed here but can be found in the sdfconf manual (see the Supporting Information).

This is how metastatements work:

- Sdfconf reads a set of molecular structures from an SDfile.
- Structures are divided into groups
  - Default by molecule name, but this can be changed to any metafield.
- Each structure of each group is given a conformation number.
  - Conformation number can also be read/written from/to a file.
- Now each structure can be identified with unique pair of molecule name and conformation number.
- Metadata is stored for each structure and can be added
  - To the whole file, each molecule, and conformation
  - By groups, like name
  - By conformation
  - By atom
- Metastatement is an expression applied for the whole file but might mean slightly different things for each structure.
  - For example, different molecules might have different atoms marked for some specific purposes.
  - Functions might give values in different scopes.
    - For example, “score/mmax(score)” norms each structure by the maximum score for each molecule group, and all differently named molecule groups have at least one structure returning 1.0 for a given metastament.

Sdfconf installers can be downloaded from www.utu.fi/sdfconf for Linux (rpm) and windows. Furthermore, a python wheels package is also available, which can be installed with pip to most platforms.

Future goals include but are not limited to

- Migration to Python 3.8
- Further performance optimizations
- Selection of atoms of molecules by structure, e.g., by SMILES string
- Storage of nested metadata, e.g., lists in a dictionary

## Data Set

As an example data set, we used compounds from the ChEMBL^3,4^ database for estrogen receptor alpha. This data set is purely for demonstration purposes, and we do not intend to produce novel scientific information. Therefore, details of the set are not of interest here.

### Ligand Selection and Preparation

All estrogen receptor alpha compounds with reported IC_50_-value were downloaded (10th of September 2019) from the ChEMBL database. They were sorted according to the activity data in ascending order so that only data with exact IC_50_ values were considered (marked with Standard Relation = ), and then, every 25th compound was extracted into the data set. Extracted ligands were prepared for molecular docking using LigPrep in Maestro (Schrödinger Release 2019-3: LigPrep, Schrödinger, LLC, New York, NY, 2019). In LigPrep, the OPLS3e^5^ force field was used, and ionization for compounds was done at pH 7.4 ± 0.0 with Epik.^6,7^ From 3901 compounds, a set of 99 compounds was formed.

### Protein Structure Selection and Preparation

The crystal structure for estrogen receptor alpha (ERα) was downloaded from the Protein Data Bank (PDB; www.rcsb.org).^8,9^ The chosen structure, PDB-code 1XPC,^10^ has a 1.6 Å resolution and a dihydrobenzoxathiin, selective ERα modulator, bound. Using Bodil Modeling Environment,^11^ the bound ligand and water molecules were removed. Hydrogens were added by using REDUCE v. 3.23.130521.^12^

### Molecular Docking

Ligands were docked into prepared protein structure using Plants v. 1.2.^13,14^ In Plants docking, the chemplp scoring function was utilized. The docking speed setting was set to speed1, meaning the highest reliability but the slowest performance. Rmsd value for clustering similar structures was 2.5 Å. Ten conformers per compound were written out. Finally, the docked conformations in mol2-format were converted to sdf-format using openbabel.^15^

Preparation and docking of molecules was not performed with the intention of generating the best possible docking results but merely to generate a diverse set of protein–ligand complexes. In a practical study one could produce only one score and corresponding complex for each molecule, but here we produce a larger number and select the preferred conformation at a later stage.

## Examples

Here, some walk-throughs of how to utilize sdfconf are given. You can find all example files in the Supporting Information. Used handles and their abbreviations are explained shortly in comment lines starting with # after the command. Each handle is explained once. Note that all commands are single line and line breaks are due to limitations of print layout.

### Importing External Data to Metadata (by Conformation)

In this example, we begin with an SDfile (docked_structures.sdf), which contains molecular docking results (in our case, ten conformers/ligand from Plants molecular docking). First, information about the original compound name (correspondingNames.csv) and the Plants docking score (ranking.csv) from csv-files are merged to SDfile (1). Sdfconf can import new metadata into an SDfile from another SDfile (combine) or from a comma- or tab-separated value file (csv) (import). In both cases, only data with the same names in both files will be imported. Names may also include conformation numbers. If conformation numbers are not provided, data will be applied to molecules with the same name. In contrast, if the conformation numbers are provided, data will be added to molecules with the same name and conformation number. In essence, one may import both molecular and conformational data at the same time by providing conformation numbers for conformational data and only molecule names for molecular data. Note that sdfconf does not understand the context of one molecule by structure but by the name of the molecule. Metadata may also be exported from SDfile to a csv-file:
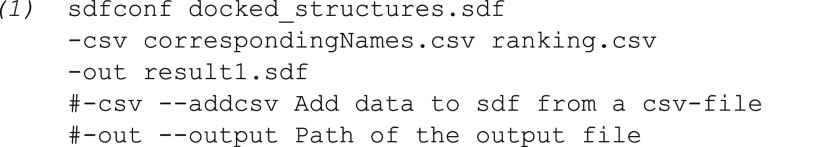


### Renaming Structures

As a default, Plants adds entry and conformer information to all compound names (e.g., CHEMBL100231 → CHEMBL100231_entry_00042_conf_01) and writes out a file called correspondingNames.csv, which contains information about the name given by Plants and original compound name (see correspondingNames.csv: PLANTS_LIGAND_DESCRIPTION, OLD_LIGAND_DESCRIPTION, respectively). This information was imported to an SDfile in the previous example (1). Next, the molecular name given by Plants from the header to new metadata (called plants_id) is stored, and compounds according to the original compound name are renamed and stored to a new SDFile (result2a.sdf; 2).

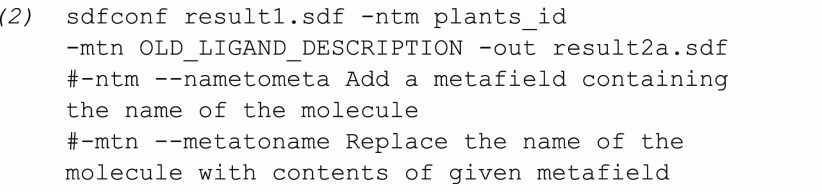



### Editing Metadata

Sometimes files like correspondingNames.csv are not available, and it is preferred to edit existing information to create new metadata. For example, here, new metadata called “real_id” is created. This data is derived from “plants_id” by removing a regular expression that describes details added by Plants (3). Essentially, the result is the same as in the previous example. Note that subtraction of strings is always considered a regular expression operation.

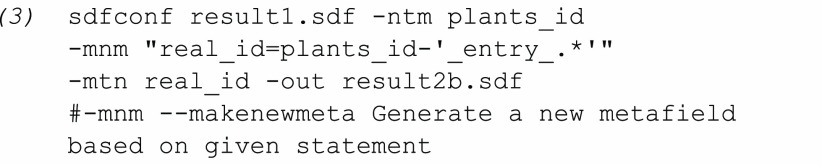



### Listing and Removing Metadata

In this example, the main interest is in storing the docking score given by Plants (imported in the first example from ranking.csv). Notably, ranking.csv contains plenty of additional information, which is here used as an example of data that is not kept in the SDFile. With the following commands, it is possible to list the metadata found in SDFile (4) and keep only the wanted metadata. This can be done either by selecting the data that is not kept and then removing metadata by name (as in result3a.sdf; 5) or removing all metadata except those listed in the command (as in result3b.sdf; 6).

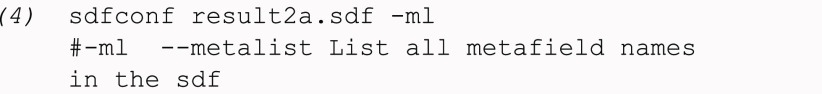

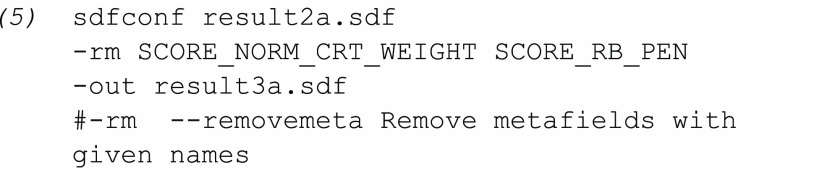

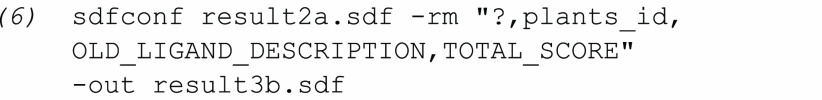







### Importing External Data to Metadata (by Molecule)

In the first example, data that was related to different molecular conformations was imported. Now, data that is not conformation-specific data but is related to molecules are added. In this example, experimentally measured activity values (IC_50_ value in nM for ERα; activity.csv) are added (7). Essentially, the structure SDfile (e.g., result3b.sdf) that is used has multiple structures with the same name, which are conformations of the same molecule. Now, the data will be added to all compounds having the name specified in activity.csv (several structures in SDFile). To make such an addition possible, renaming the compounds like in the previous example is important.

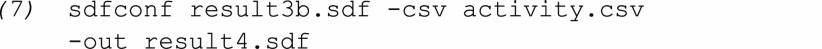



### Plotting Data: Scatter

**Figure 3:**
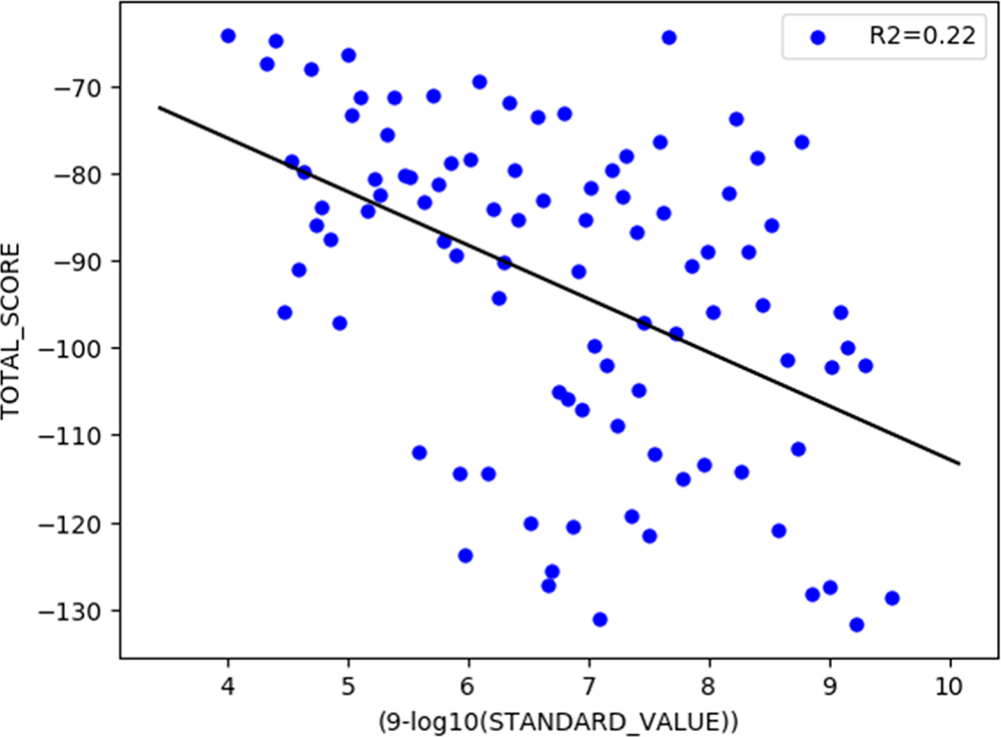
Example scatterplot.

Quite often, quick visualization of the data is desired. Next, a scatter plot with a trend line of activity data versus Plants docking score (TOTAL_SCORE) for the best-ranked conformation (the minimum value) is created (8). The activity data is imported as IC_50_ in nM, which is converted to pIC_50_ (negative logarithm, i.e., pIC_50_-value 9 equals IC_50_ of 1 nM). As a default, sdfconf prints the SDfile contents to standard output, which in the previous command is directed to a new file with an “-out” handle. Here, the printing of sdf file contents is altogether prevented with the “-dnp” handle, and only the figure is printed and saved. We also use a few special commands to “-sca”, trend draws the trend line and R2 value, while save saves the figure as a file in addition to the normal printing. The resulting image can be seen in Figure 3.

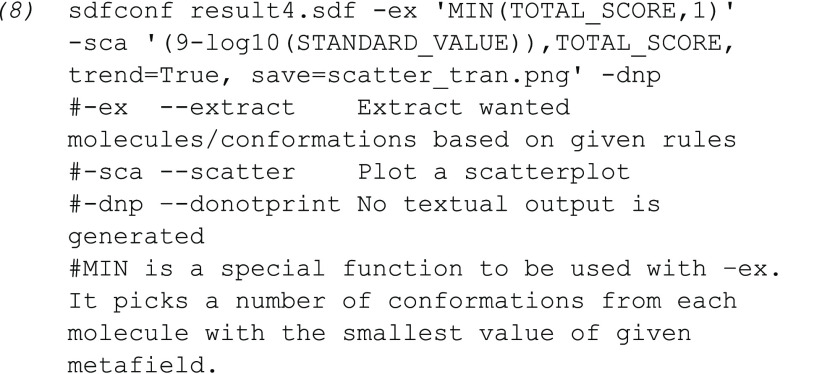



### Plotting Data: Histogram 1D

**Figure 4:**
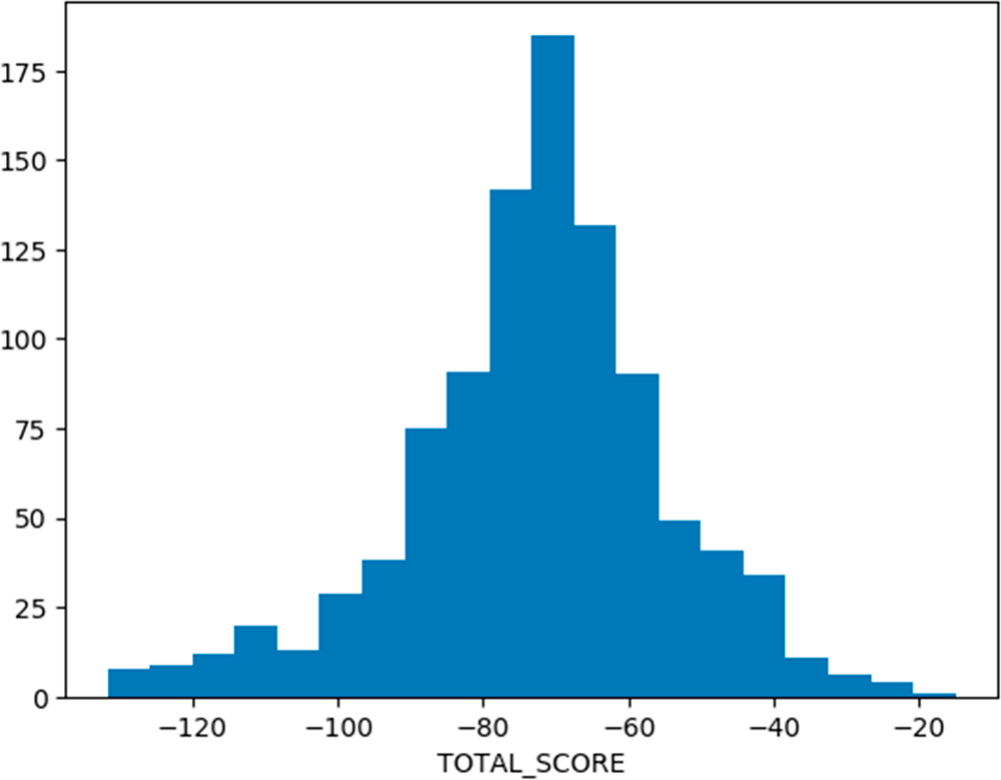
Example 1D histogram.

Here, a 1D histogram of Plants docking score for all conformations is plotted Figure 4. Values are divided into 20 bins (9).

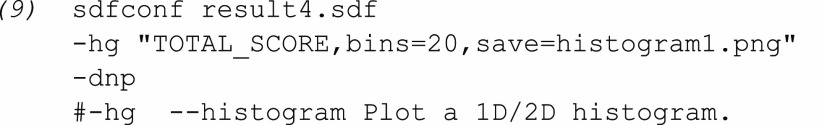



### Plotting Data: Histogram 2D

**Figure 5:**
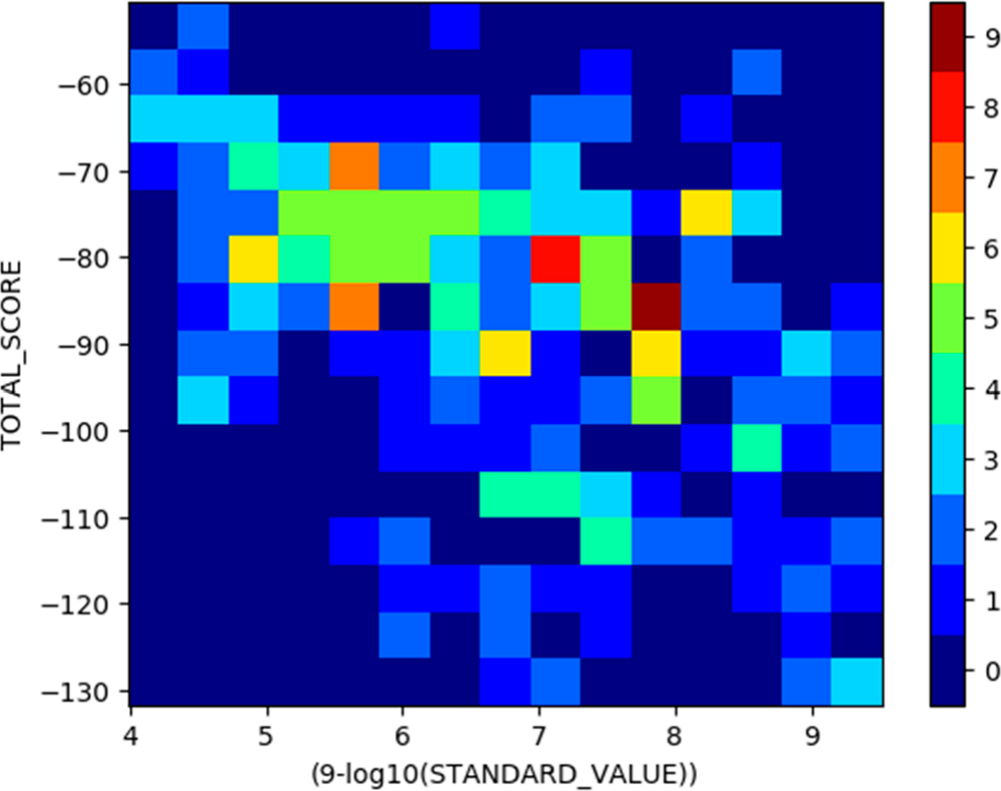
Example 2D histogram or heatmap.

Also, a 2D histogram, or heatmap, can be plotted: In Figure 5, Plants docking score versus calculated pIC_50_ for best 30% of conformations for each molecule is plotted (10). Values are divided into 15 × 15 bins.

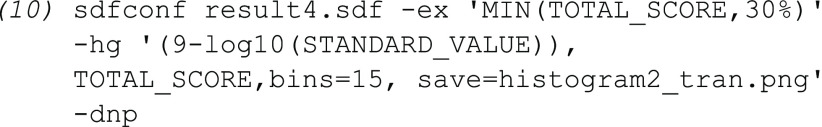



### Selection/Elimination of Conformations Based on Specific Coordinate Point

In the ERα binding site, there is one specific glutamic acid (Glu353), to which quite many of the ERα ligands form a hydrogen bond. Here, the ligand conformations that have the potential to form a hydrogen bond with Glu353 with a hydroxyl group are searched. Accordingly, the search of both oxygen and hydrogen atoms (approximating a hydroxyl group) from 2.5 Å distance from GLU353:OE2 using its *XYZ*-coordinate point (24.31, −3.29, 25.59) is performed. For clarity, example commands are given in parts 11–14. First, distances of all atoms to our point-of-interest are calculated and saved into metadata called glutadist (11). Handle “-ig” sets ignorelist to empty; by default, it is “H”; therefore, all operations ignore hydrogens, but now they are included. Note that the glutadist-metadata consists of specific information for each atom in each molecule conformation.

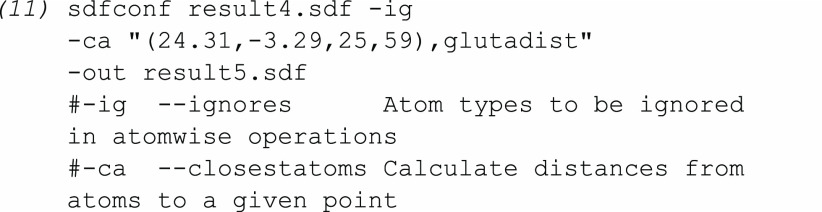



In the second step, the atom types of those atoms that reside 2.5 Å or closer to our point-of-interest are defined (12, 13). Internal function “confcol(4)” picks values from SDfile atomblock column 4, which stores atom elements (see Figure 2) and then saves them to new metadata “atom_types”. With “glutadist(≤2.5)”, we pick entries from metadata “glutadist” with values (distance) that are 2.5 (Å) or smaller and store them as new metadata “close_atoms”. Next, “close_atoms{}” picks just the keys, or atom numbers, of “close_atoms” without values and stores them as metadata “close_atom_nums”. With “atom_types(close_atom_nums)” we pick entries from “atoms_types”, by atom numbers in “close_atom_nums”, these are stored as metadata “close_types”. Eventually, “close_types” stores atom numbers and types of atoms in the range of 2.5 Å from the given point.

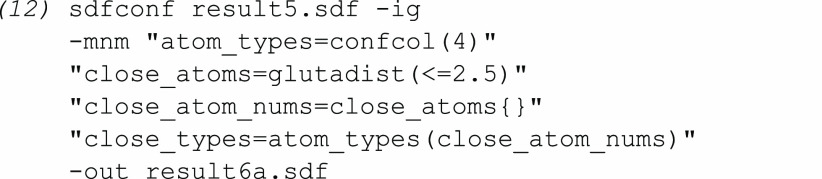

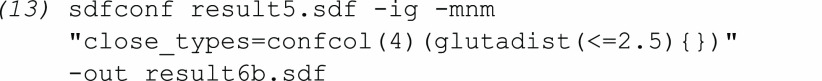





Finally, we pick conformers that have both oxygen and hydrogen close to the point-of-interest (14).

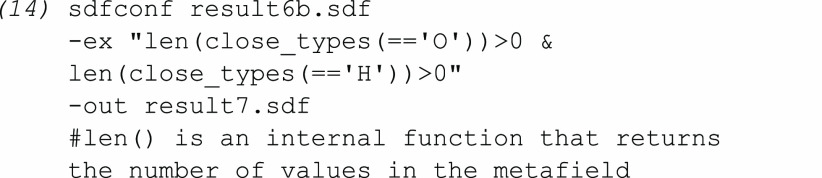



### Importing Data from Another File Format

In the previous example, we used specifically oxygen and hydrogen to define atoms of our interest. SDfile does not typically consist of information about more specific atom typing, and yet it might be valuable to identify atoms with specific properties in a given 3D space. For example, it might be of great interest to identify any hydrogen bond donor in the hydrogen bonding distance to glutamic acid. Such data could be, e.g., atom types O.3, N.4, N.3 in mol2-format.

Here we convert result6b.sdf to result6b.mol2 using openbabel.^15^

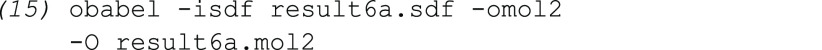



Now we can pick Sybyl atom types from the said file as metadata named “sybyl”. Then we proceed to pick only atoms close to point-of-interest using previously derived “close_atoms” and create metadata “oxytest” and “hydtest”, containing only atom types “O.3” (oxygen sp3) and “H”, respectively. Finally, we can look for conformations containing both. This example (16) and the previous example (14) yield the same result.

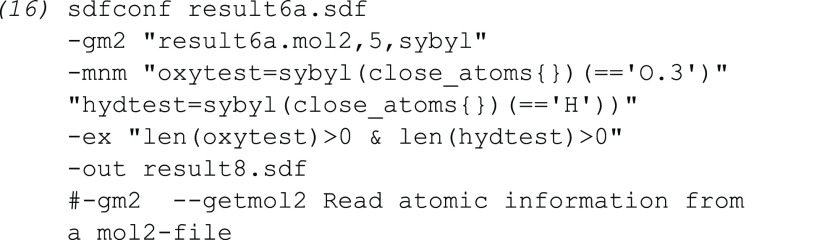



More examples of how sdfconf has been utilized in research can be found from recent studies.^16−19^ For example, classification of molecules/conformations based on any property and importing and exporting data into metafields have been used extensively. Kurkinen et al. used sdfconf to calculate the average geometric centroids for the top-ranked docking poses.^17^ Jokinen et al. and Juvonen et al. employed sdfconf to filter docking poses to find those conformations that have the potential to form a hydrogen bond with a certain atom in amino acid. This was done by measuring distances between chosen atoms.^16,18^ Rauhamäki et al. used sdfconf to generate a simple pharmacophore filter to refine docked conformations. Sdfconf was used to select only conformations without hydrophobic groups in proximity of a certain side chain atom.^19^ In addition, sdfconf has been utilized to search sites of metabolism prediction (unpublished data). Moreover, sdfconf has facilitated data handling in many projects, where plenty of metadata or a multitude of conformations of small molecules is concerned.

## Conclusions

Sdfconf is not a premade solution to any specific scientific or logical problem, but instead, it provides a flexible set of available tools to solve various problems in “data logistics”. Although the authors sincerely hope that future developments by users are made available to other users that is not required by the license.

## Data and Software Availibility

Sdfconf is licensed with MIT/expat license. It is free to use for all users. Binaries and source code are available at www.utu.fi/sdfconf. It is possible to modify and redistribute sdfconf from source code under MIT/expat license, which requires one to retain the original copyright notice.

Example data is available as Supporting Information and is sourced from public data.

## Supporting Information Available

All files used or generated in the examples, excluding figures. This includes molecular structures and used tabular data (ZIP)
Manual for sdfconf, which explains the inner workings of sdfconf in greater detail. It also describes syntax for metastatements, available functions, and handles, etc. (PDF)
