# Supplementary material for: Sdfconf: A Novel, Flexible, and Robust Molecular Data Management Tool
Source: J Chem Inf Model. 2021 Dec 21;62(1):9–15. doi: 10.1021/acs.jcim.1c01051 (PMC8757437; doi:10.1021/acs.jcim.1c01051)
Supplement: Supplementary file 2 — ci1c01051_si_002.pdf [file ci1c01051_si_002.pdf]

sdfconf manual

The metastatement guide

For sdfconf v0.8.37

Contents

Introduction 5
How to use sdfconf 5
Passing parameters from command line 6
Order of execution 6
Installation 6
Metadata 6
Datatype 6
String 6
Integer 7
Float 7
Datastructure 7
Single 7
List 7
Dictionary 7
Metastatements 7
Calling stored meta 7
Reserved names 7
(Partly)nonexistent names 8
Names with operators 8
Literal values 8
Parenthesis 8
Parenthesis () 8
Square brackets [] 9
Curly brackets {} 9
Quotation marks ‘’ “” 9
Operators 9
Summation and subtraction + - 9
Product and division * / 10
Remainder % 10
Power ** 10

Joining and cutting ++ --........10
Functions........10
Sorting........10
Statistical........10
Mathematical........11
Other........11
Comparisons........12
Metaslicing........12
(Non/In-)Equality == != >= <= < >........12
Metacomparison........12
(Non-)Equality == !=........13
Inequality >= <= < >........13
Regular expressions........13
Features........13
Conformation numbers........13
Chopping........14
Import Export........15
Combine........15
Cut........15
Distance calculation........16
Overlapping........16
Sorting........16
Config-files........17
Atom removal........17
Mol2-extraction and -injection........17
Plotting........17
Verbose........18
Examples........19
closestatoms, allcombine, getcsv, conftometa, makenewmeta........19
Use as a library........20
Internal structures........20
runner........20
sdf........20

mol2 21
functions 21
Findable 21
Appendix 22
Appendix A 22
Appendix B 2
Appendix C 2

Introduction

sdfconf is used to do things that are otherwise difficult to do. The motivation for the sdfconf was the absence of software tools that could store and analyze multiconformational data for small molecules. Accordingly, the choice for the biologically relevant ligand conformation within its binding site in protein was based on single data, or command line argumentation.

sdfconf is a software package written in python. It's purpose is to enable easy, flexible and versatile way to handle, manipulate and analyze conformational data of molecules. It mainly works with SDfiles (.sdf), but some operations also work with .mol2 and .pdb files. In practice, any data stored into mol2 can be imported: Atom types, partial charges etc.
Main feature of sdfconf is using mixed, molecule- or conformation-wise data from mixed sources to organize and select molecules. It can present results as SDfile, two flavours of .csv or simple counts of remaining molecules. It can also plot histograms in 1D and 2D (heatmap) or scatter plots.

How to use sdfconf

In general, sdfconf is rather simple and straightforward to use when considering the variety of possible operations. Generally complex results require complex commands. When using sdfconf, the default help offers useful explanations for different options.
All functions of sdfconf can be divided to a few important categories. The categories and their respective options are:

- input*: SDfile to manipulate/analyze with the software
  - input
- augmentation: Addition of data from a number of sources
  - combine, allcombine, addcsv, addatomiccsv, getmol2
- calculation: Calculation of information from existing data
  - closestatoms, closeratoms, makenewmeta, addescape, addinside
- conformations: manage conformation numbers
  - conftometa, conftoname, removeconfname, removeconfmeta,
- manipulation: Changing and removing of data
  - nametometa, metatoname, changemeta, stripbutmeta, sortorder, removemeta, pickmeta
- chopping: Using the extract option to remove undesired conformations
  - extract, allcut, cut
- plotting: Plotting of histograms
  - Histogram, scatter
- output type*: What to output? sdf, csv, conformation counts, metanames, nothing?
  - getcsv, getatomcsv, metalist, counts, donotprint
- output*: Where to output? Overwrite, file, screen?
  - output, overwrite
- output options: Make folders or multiple files?
  - split, makefolder
- options: Various options on the run, like verbose, ignores and proportion.
  - verbose, ignores, proportion, config

- injection: Inject data to external files
    - putmol2

These are just arbitrary groups of actions that aren't explicitly present in the program, but make the software more approachable. Note that not all runs have all of these, only those marked with * are present in every run in some form.
Important and complex options are discussed in more depth in section Examples.

Passing parameters from command line

Many of sdfconfs

Order of execution

When calling sdfconf, options are executed in following order:
Verbose, nospam, input, molgrouper, confgrouper, conftometa, conftoname, nametometa, removeconfname, removeconfmeta, ignores, cut, allcut, combine, allcombine, addcsv, addatomiccsv, getmol2, addescape, addinside, config, closestatoms, closeratoms, changemeta, makenewmeta, sortmeta, stripbutmeta, proportion, sortorder, extract, metatoname, removemeta, pickmeta, putmol2, histogram, scatter, getcsv, getatomcsv, metalist, counts, donotprint, donotplot, split, makefolder, output, overwrite.
Arguments passed on comand line take place in this order. Options used in config files are interpreted in order of appearence, at time when 'config' is executed.

Installation

Currently, there are packages as rpm for linux and executable installer for windows. Universal python wheel distribution and source distribution are also available.
To use rpm distributions, use package managers available for your linux distribution. For wheel distribution as well as installing source distribution, you need python setuptools. Wheel may be installed using python package installer pip. To install from source, after extracting the tar, change directory to package root and run python setup.py install. Note that there is no uninstaller for source installation. Using windows installer should be rather straightforward.
After installing sdfconf, command "sdfconf" should be available.
sdfconf may also be used without installing by running <package_folder>/bin/sdfconf
sdfconf requires following packages to be installed: numpy (1.7.1 or later) and matplotlib (1.4.2 or later).

Metadata

sdfconf can store, manipulate and utilize molecule-, conformation- and atom-wise data in a way referred as metadata. All stored metadata has a name and some sort of value. It can never be without value, if it is, it does not exist at all. Temporary metadata may exist without a name.

Datatype

sdfconf supports three basic types of data: string, integer and float.

String

Metadata may include strings. They can basically include anything.
Internal python type is "str"

Integer
Integer numbers. Whole numbers, positive and negative.
Internal python type is “int”

Float
Floating point numbers. All real numbers, positive and negative.
Internal python type is “float”

Datastructure
In plural datastructures all entries must have the same datatype. If a list includes [1, 2, 3.1], datatype is float. If a list includes [1, 3, oxygen], type is string. Casting to different types is done automatically.

Single
Singular data. One number or string. Effectively is a list with one item.

List
List of data. A number of data entries under one name. For example list of integers might express certain desired or undesired atom numbers.

Dictionary
Dictionary is a list of paired data. Every entry has a key and a value. For example atom number and partial charge of said atom.
Internal python type is “collections.OrderedDict”.

Metastatements
Many functions of sdfconf are based on so called metastatements. They are logic/arithmetic statements that are used to define properties for molecules and conformations. These are in many ways the point of sdfconf and are worth learning, despite being quite difficult at first. Experience in scripting languages like python or numeric calculation software like matlab may help.

Calling stored meta
One may refer to any metadata stored in molecule by simply it’s name. For example if SDfile includes lines

```
>  <ID>
benzene
```

one would refer to said meta by typing “ID” and it would return singular string “benzene” in case of said conformation. On other molecules it would return the corresponding value.

Reserved names
All function names specified later in Functions section are reserved. Creation of such metadata is possible, but it’s impossible to refer to them in metastatements. There are also certain names that are created by some routines of sdfconf, but they are just conflicting metanames.

(Partly)nonexistent names

If a query with metaname that doesn't exist on any or some conformations, nothing is returned for these conformations. Usually this leads to warnings about non-created metadata. If used in logical comparison, such values result in logical false result.

Names with operators

If metaname includes an arithmetic operator, sdfconf tries to interpret it. For example if a conformation has metas with names "a", "b" and "a-b", making a query "a-b" would actually result in "a"-"b". Operators may be escaped by adding a preceding backslash, or as in previous example "a\-b".

Literal values

Numbers in statements generate a metadata including that singular number. Strings may be generated by enclosing a string in quotes. More on quotes in section "Quotation marks".

Parenthesis

In following sections let's assume following metas:

- meta1 = {1:1.1, 2:2.2, 3:3.3, 4:4.4, 5:5.5}, dictionary type
- meta2 = [1,5,3], list type
- meta3 = {O:1, N:2, X:3, B:3,C:8}, dictionary type

Parenthesis ()

Parenthesis have four meanings in sdfconf. First one is to prioritize operations, second is to slice selected parts of dictionary type meta, third is to make logical type slices from any meta and fourth is as enclosure for function arguments.

Prioritizer

Used as in standard arithmetic. For example (1+2)*3 yields 9 while 1+2*3 yields 7.

Dictionary slice

It is possible to pick selected parts of dictionary by slicing it with explicit keys or meta of list type. For example meta1(meta2) would yield {1:1.1, 5:5.5, 3:3.3}. meta1(5) would yield {5:5.5}. Note that in first example values of meta1 are returned in the order of matching keys in meta2.
Functionality changed in 0.8.36. In addition to previous examples, meta3(meta1) yields {O:1.1, N:2.2, X:3.3, B:3.3}.

Logical slice

Parenthesis may be used to slice parts of meta by comparing values of its elements to some other value(s) of some other meta. It's distinguished from other parenthesis by adding a comparison operator as the first element in the parenthesis.
Let's take meta1 and meta2. Now meta1(>4.3) would yield {4:4.4, 5:5.5}, so with dictionary types, comparison is made with values (and not with keys). In other hand meta2(<=3) would yield [1,3]. Also meta2(meta2(<=3)) would work and yield {1:1.1, 3:3.3}. Also equality/inequality between strings is possible.
For more about logical metaslicing, look section "Comparison, metaslicing".

Argument enclosure

There is a number of builtin functions that may be used with syntax “func(meta)”, for example “sum(meta2)” would yield 9, while “max(meta1)” would yield 5.5.
More in section Functions.

Square brackets []

Square brackets have only one meaning, which is slice values from meta. One might use plain numbers, or python style indexing. With list types, action is quite simple, but in case of dictionaries, one picks from values of dictionary. For example, meta2[0] = 1, meta2[1:2] = [5,3], meta1[:2] = [1.1,2.2].

Curly brackets {}

Curly brackets can be only used with dictionary style meta. They have two meanings.

Slicing dictionary keys

First use for curly brackets is slicing keys from dictionaries. They work just like square brackets in slicing, but return dictionary keys instead of values.

Logical slicing by keys

Secondly curly braces can be used in logical slicing of dictionaries by keys. They work like parentheses in logical slicing, but comparisons are made against dictionary keys.
For more about logical metaslicing, look section “Comparison, metaslicing”.

Quotation marks ‘’ “”

Queries inside quotation marks will always generate a new meta including a literal string inside the quotes.

Operators

Operators can be used to make combinations of different meta.
With list type meta, it is only possible to make operations with other lists of same length or singular meta. Operations between applicable lists makes operations between single items of the lists, in the order of appearance. If one of the two meta is singular, it affects all elements of the list.
When doing operations with dictionary type meta, only operations with singular data or other dictionary type meta are possible. With two dictionaries operations are performed between elements of each dictionary with same keys. Keys without a pair in other list are omitted. Keys of resulting meta are in the order of the first dictionary. If one of the meta is singular, it affects all values of the dictionary.
Operations between singular meta are straightforward operations between two values.
In all of the cases, singular meta may be a single type meta or list with one value.

Summation and subtraction + -

Basic summation and subtraction. With numeral values, if some of values in resulting meta are of float type, all values are cast into float type.
In the case of string type meta, summation concatenates strings. In case of subtraction of strings, last string is turned into regular expression and matching parts of the first string are removed. For

example “benzene{[01]}” - “\{\[\d{2}\]\}$” yields “benzene”. Regular expressions use python re module.

Product and division * /

Product and division are standard arithmetic operations. They are only possible between numeric meta.

Remainder %

Remainder is the arithmetic division remainder. For example [7,11] % [3] yields [1,2]. It is only possible between numeric meta.

Power **

Power is standard arithmetic operation. It’s possible only between numeric meta. For example [2, 3]**[3] yields [8, 27]. It is only possible between numeric meta.

Joining and cutting ++ --

Joining and cutting are used to combine two metadata into one.
With list and singular type meta joining just concatenates lists together. Cutting in lists on the other hand removes all items from the first list that are present in the last one.
In case of dictionary type meta, joining adds keys from last meta to first, that are not already present. Cut between dictionary type meta removes key from first that are present in the last.

Functions

Functions are called by “func(metastatement)”. They return a meta themselves so the are also a metastatement.

Sorting

Sorting functions change the order of elements in meta. Sorting of lists is very straightforward and with dictionaries, keys are sorted based on the values.

asc

asc sorts meta to ascending order.

des

des sorts meta to descending order.

Statistical

There are two kinds of statistical functions. max, min and avg return the desired result of single meta, but mmax, mmin and mavg return the result for desired meta from the whole set of conformations with the same name.

max

max returns the maximum value of argument statement for each conformation.

min

min returns the minimum value of argument statement for each conformation.

avg

avg returns the average value of argument statement for each conformation.

mmax

mmax returns the maximum value of argument statement for the set of conformations with same name.

mmin

mmin returns the minimum value of argument statement for the set of conformations with same name.

mavg

mavg returns the average value of argument statement for the set of conformations with same name.

gmax

mmax returns the maximum value of argument statement for the whole file.

gmin

gmin returns the minimum value of argument statement for the whole file.

gavg

gavg returns the average value of argument statement for the whole file.

Mathematical

sum

sum returns the sum of values in single meta for each conformation.

prod

prod returns the product of values in single meta for each conformation.

log10

Returns the base-10 logarithm for all values of meta

ln

Returns the base-e natural logarithm for all values of meta

Other

len

len returns the length of meta produced by argument metastatement. length means number of elements in one meta. For locally non-existent meta len returns 0.

rdup

rdup returns given metastatement, but removes duplicates from the output.

confcol

confcol returns an asked column of conformation atom block for each conformation. Returned meta is a dictionary type meta, whose key is atom number and value is requested column for said atom in atomblock. Argument should be an integer, when one may obtain numeric coordinates,

etc. If argument is a list, one gets a strings for each atom combining the requested columns. Usually motivation to use confcol is to obtain atom types, chiralities, etc.

getmeta

Allows user to call a stored meta with same name as given string type meta. Enables user to call “dynamic” metanames.

str

str casts meta from given statement to string. Cast is made for element values for plural meta. Required if one desires to concatenate string and numeric type meta.

dictmirror

dictmirror reverses keys and values of dictionary type meta. For example meta {1:X, 2:A, 3:B, 4:X} dictmirror(meta) yields {X:1,A:2,B:3}.

construct

construct generates a new meta from a string in a similar way to how metas are generated from files. For example construct(“1:3.3,2:7”) yields a dictionary type meta {1:3.3,2:7}.

Comparisons

Metaslicing

Metastatements may include traditional (python-style) index-based slicing of lists and dictionaries. But one may also perform logical slicing of meta based on the values of the meta.
In metastatements also the slicing values are cast into a meta data and they might be simple singular, explicit numeric values. In this case all values in meta are compared to the slicing value and only those fulfilling the comparison condition are selected to the new, sliced meta.
If slicing values are not singular, in case of list meta, slicing value must be of the same length as the meta to be sliced. Then the values of two lists are compared to each other in the order of appearance. In the case of dictionaries, if slicing meta is not singular, it must be an dictionary type and must contain all the keys present in the meta to be sliced. Then the values of the two dictionaries are compared key-wise.

(Non/In-)Equality == != >= <= < >

The comparisons are standard equality, non-equality and inequality. They are all treated the same way, except for that equality and non-equality can perform string comparisons with regular expressions, which are described in section Regular expression.

Metacomparison

Metacomparison is not part of metastaments per se, but they are rather comparisons that contain two metastatements separated by comparison operator. As opposed to metaslicing comparisons are always related to certain meta generated by a metastatement.
The purpose of metacomparisons is to select desired conformations from a group of molecules and conformations.

Quite unorthodoxically, metacomparisons on non-singular meta are NOT symmetric. Equality and non-equality comparison are symmetric, but inequalities are not. Symmetry is more described in specific sections of equalities.
In general it is impossible to compare dictionaries with non-singular, list type meta.
Metacomparisons are used for example by --extract and --proportion options.
It is possible to invert the positive and negative results of a comparison by adding “- “ to the beginning of comparison string. It’s possible to explicitly use the default behaviour by adding “+ “ to the beginning of the comparison, but it’s not required.

(Non-)Equality == !=

When comparing meta for equality or non-equality, a positive result is triggered if even a single pair in two meta fulfils the condition. Therefore, not all elements of meta are required to be (non-)equal.

Inequality >= <= < >

When comparing meta with inequalities, one must note that comparisons are not symmetric. Therefore a<b might yield different result that b>a. When meta are non-singular, the logic for positive result is the following: “At least one element on the right hand side must fulfil the condition for all elements on the left hand side.”
For example 5>[3,5,7] is positive because 3 on the right side is smaller than all elements on the left side.
On the other hand, [3,5,7]<5 yields negative result, because not all on the left side are smaller than at least one on the right side.
Again, [3,5,7]<[5,10] yields positive, because all one the left side are smaller than one (10) on the right side.

Regular expressions

One may create strings that are treated as regex in comparisons. Such meta value should be of form “REGEX:yourregex”, where yourregex is the desired regular expression. REGEX must be in capital letters. Regex comparisons are only intepreted in equality and non-equality comparisons.

Features

Conformation numbers

--conftometa
--conftoname
--removeconfname
--removeconfmeta

sdfconf always assigns conformation numbers to all molecules. Single conformation is identified by it’s name and conformation number, therefore conformations with different names may have same conformation numbers. sdfconf may store conformation numbers explicitly in two ways. First way is in the name field of the molecule, this way the conformation closed in curly and square

brackets. For example “phenytoin{[01]}”. If SDfile loaded into sdfconf has these kind of names, the given conformation numbers are used. The second way to store conformation numbers is in a metafield “confnum”. If conformations have a metafield with this name and it includes singular value, it is used as conformation number. If molecule has both values, it rises a warning message and conformation number in name is used.

Chopping

--extract metachop

Extract is used to select or drop conformations from SDfile. metachop can have a few different forms:

- metacomparison: metastatement logic metastatement
    - my_docking_score >= 100
        - metafield “my_docking_score” has value equal or greater than 100
    - my_docking_score >= 0.5*mmax(my_docking_score)
        - metafield “my_docking_score” has value at least half of the greatest value of “my_docking_score” occurring in conformations with same name
    - 5.0 < poi_distance(atoms_of_interest)
        - “poi_distance” is created with “closestatoms” option. “atoms_of_interest” is list of atom numbers that are somehow interesting. Conformation that have at least one interesting atom with distance less than 5.0 to point of interest are picked. (Note that if comparison was other way around, conformations whose all interesting atoms were closer than 5.0 to poi would be picked.)
- part: mima(metastatement, amount)
    - MAX(my_docking_score, 3)
        - Three conformations with largest values of “my_docking_score” are picked
- unique: !metastatement
    - !best_atom_from_test
        - “best_atom_from_test” is single atom number. Molecules with duplicate value of this atom number are dropped.
    - !asc(poi_distance(atoms_of_interest)){0}
        - conformations with unique closest atom number to poi are picked

With metacomparison one defines to metastatements and compares them with selected logical operator. Those that yield positive result, are picked.
With part, one uses one of two functions, MAX or MIN to pick conformations either with largest or smallest values of given metastatement. The amount is either a absolute number of conformations or percentage of conformations.
Unique removes molecules that have duplicate values of given metastatement. The first occurrence of certain value gets picked.
Note that you may give multiple, separate, logical statements resulting in “logical and”-like behaviour. You may also combine multiple logical statements with boolean operation “and” and “or” with “&” and “|” respectively.

Import Export

--addcsv path [,molcol=<column>] [,confkey=<column>]
--addatomiccsv path [,molcol=<column>] [,confkey=<column>] [,atomnnumber=<column>]
--getcsv metalist
--getatomcsv metalist

sdfconf can import new metadata into a SDfile from another SDfile (combine) or a comma- or tab-separated value file (csv) (import). In either case only data with matching names in both files will be imported. By default, names should be in the leftmost column. Names may also include conformation numbers which are discussed in section [Features/Conformation numbers]. Conformation numbers may also be given in specified column. If conformation numbers are not provided in imported data, data will be applied to molecules with the same name, where as if they are provided, data will be applied to molecules with same name and conformation number.
When importing from normal csv-file, single values may appear as they are. In cases of lists and dictionaries, data must be in quotes (“ or ‘), with values separated with comma or semicolon (, or ;). Dictionaries include a key and a keyed value in place of a single value. Key and keyed value are separated with a colon (:). In case of atomic csv-files, one column must specify atom numbers, which by default is column named “atom_number”.
Atomic csv-files are used to import atom-wise data. Single line should specify molecule name, possible conformation number and atom number. All atom numbers for single conformation are collected and dictionary type meta is generated for the conformation.
Metadata may also be exported to a csv-file. Format is similar to that of imported csv-files. One should provide a string containing a list of metanames (separated by ‘,’). Note that these cannot be metastatements. In the list of metanames ‘?’ means ‘all but’. For example ‘?,mydockingscore’ would return csv with all metadata, but not ‘mydockingscore’.
It is also possible to export atomic csv file, which has a line for each atom for each conformation. Atom number is specified as first column and information in dictionaries is applied to them atom number wise. Other meta is applied to all lines. Currently it’s not possible to read such import such .csv-files.

Combine

--combine path_to_sdf
--allcombine path_to_sdf

Combine tools import meta data to SDfíle from another SDfile. --combine requires matching names and --allcombine also requires matching conformation numbers.

Cut

--cut path_to_file
--allcut path_to_file

Cut tools remove molecules or conformations from SDfile. The standard one requires matching names and conformation numbers, while the all variant requires only matching names. It accepts an SDfile or csv-file as argument. csv-file might be one column, i.e. namelist text file.

Distance calculation

--closestatoms (xx, yy, zz) [,name] [,interests=value]
--closeratoms (x,y,z),meta

Distance calculation tools give information about distance of atoms to points of interest. There are two separate tools.
First is closestatoms, which requires one point of interest (x,y,z) and then calculates distances from that point to all atoms of conformations and adds a meta with given name. Meta is of dictionary type and includes distances as values by atom numbers as keys. If metastatement interests is specified, distances are calculated only for atom numbers specified in it. If no name is given, meta is named “Closest_atoms”.
Second method is closeratoms that in addition to point of interest requires meta that defines a list of atom numbers. The method calculates which of the defined atoms is closest to the point of interest and how many atoms there are that are closer to the point of interest. In generates metafields “Closer_atoms_than_meta” and “Closest_atom_from_meta”.

Overlapping

--addinside File,range,name[,name=some][,mol=N][,max=M]
--addescape File,range,name[,name=some][,mol=N][,max=M]

Overlap tools require one given .mol2- or .sdf-file, selected molecule (actually treated as a set of points) from said file and range (sphere radius). This information is used to generate a volume consisting of spheres with given range and centers on given points. The algorithm calculates the atoms for each conformation that are either on the inside (addinside) or outside (addescape) of the generated volume. Finally atom numbers matching the query are returned. If max is given, algorithm will stop when given number of atoms are found. Value of max defaults to zero, which means infinite. Positive value of max tracks number of atoms in desired category, while negative number tracks the opposite category.
This tool can be used for example to determine if a ligand conformation as a result of docking overlaps with protein. Or it can be used to obtain information used to select conformations that lie in desired active zone of protein.

Sorting

--sortorder

Sortorder sorts the molecules in SDfile in ascending or descending order, based on values of a given metastatement. Add < (ascending) or > (descending) to the beginning of the metastatement to define the sorting order.

Config-files

--config config_file.txt

Config tool enables one to use pre-created scripts to run complex procedures in one run. The syntax for one line of config file is the following:
option_name :: argument1 ;; argument2
where

- option_name is the short or long form name of sdfconf option
- :: separates optionname from arguments
- argument1 and argument2 are separate strings including one argument for given option
- ;; separates arguments from each other

Number of arguments may be zero or more. If there are no arguments, no :: is needed. ;; is given between arguments. No ;; is needed after the last argument.
Config option is run after injection options, or in other words those that add information to run. Inside the config, options are processed in given order. Therefore one may write multiple outputs between operations, etc. Note that number of parameters might be limited by different options.
Note that in config files there is an extra option "sdf" that changes output back to SDfile. This is needed in case of multiple outputs when there are other kinds of outputs before SDfile.

Atom removal

--stripbutmeta metastatement

Stripbutmeta inteprets given metastatement and removes atoms from conformations that are not defined in the list generated by the statement. This may be used to generate files that include atoms of interest for visualization purposes, etc.

Mol2-extraction and -injection

--getmol2 pathto.mol2,column,metaname
--putmol2 input.mol2,output.mol2, column, metastatement, default, precision

One may extract data from an external .mol2-file with option getmol2. The specified mol2.-file must contain exactly the same molecules as your .sdf in the same order. The function adds a metafield with specified name, which includes atom wise data in the specified column of .mol2-file. Column indexing starts from 0.
With putmol2 one may read input.mol2 and replace data in selected column in atom wise manner with data from metastatement. Default value is used to atoms that are not specified in the metastatement. All of injected data is written with specified precision (number of decimals) to output.mol2. Therefore the original file is not altered.

Plotting

--histogram X-metastatement[, Y-metastatement][, title=figtitle][, Xtitle=x-axel[, Ytitle=y-axel]] [,args]
--scatter X-metastatement ,Y-metastatement[, group=metastatement][, trend=True/False][, legend=True/False][, title=figtitle][, Xtitle=x-axel [,Ytitle=y-axel]] [,args]

With histogram it’s possible to plot 1D histograms or 2D heatmaps. Metastatements define the values for points in x and y direction. Titles can be used to define shown names on the figure. args include other parameters into account. For both plots one may define bins variable, which defines the number of bins in the histogram. For 1D plot takes “bins=n” while 2D plot takes “bins=[n,m]”.
Scatter makes 2D scatter plots, in which you may group data points with a third metastatement. Whether or not data is grouped, one may also plot trend for the data and calculate corresponding R^2 value.
Note that also normal output is made in addition to plots. You may omit normal output with option --donotprint.

Verbose

--verbose

Verbose prints output on your run. The output is separate from sdf-related output to be written into file. Verbose output includes information on phases of running sdfconf, running times, etc.

Examples

closestatoms, allcombine, getcsv, conftometa, makenewmeta

command

sdfconf manual_test_sdf_1.sdf -aco 1A2_sub.sdf -ctf -ca ‘(49.51, 47.21, 57.36), poi’ -mnm ‘poi5=poi(:5)’ -gc ‘ID,confnum,poi5’

would yield the following output:

ID	confnum	poi5
"NDEA"	"2"	"7:3.45, 6:4.812, 1:5.2848, 3:5.5616, 2:5.7138"
"NDEA"	"1"	"7:3.7296, 6:4.9214, 3:5.2113, 1:5.477, 2:6.3938"
"phenytoin"	"4"	"15:4.2757, 19:4.4248, 12:5.2588, 16:5.4993, 9:6.2145"
"phenytoin"	"3"	"19:4.8506, 16:4.9596, 15:5.651, 13:5.8382, 12:6.4337"
"phenytoin"	"1"	"18:4.2676, 17:4.4125, 14:5.2149, 10:5.4529, 11:6.1189"
"phenytoin"	"2"	"18:4.0887, 17:4.3518, 14:4.9891, 10:5.4184, 11:5.9408"

According to order of execution, presented in General information, Order of execution, all presented options are run in certain order.

1. input: *manual_test_sdf_1.sdf* is read
2. conftometa: conformation numbers in molecule names are read and added to field “confnum”
3. allcombine: meta in *1A2_sub.sdf* are read and added name-wise (without conformation numbers) to molecules in current run
4. closestatoms: for every conformation distance of all atoms (hydrogens ignored) to given point of interest are calculated. Dictionary meta with atom numbers as keys and distances as values is added to given metafied “poi”. Dictionary is in ascending order by poi distance.
5. makenewmeta: metafield poi is read and five first appearing key-value pairs are sliced from it. This slice is added as metafield “poi5”
6. getcsv: a csv (or tab separated value) is generated with columns ID, confnum, poi5. “ID” is combined from *1A2_sub.sdf* and “confnum” is generated from molecule name by conftometa option. poi5 was made with makenewmeta.
7. As no output file is not specified, results are printed to standard output.

Note that only “input” is a positional argument. Input-file(s) must always be the first argument. All other arguments are optional and are executed in predefined order, so order in given command is irrelevant.

sdfconf manual_test_sdf_1.sdf -mnm ‘poi5=poi(:5)’ -ca ‘(49.51, 47.21, 57.36), poi’ -gc ‘ID,confnum,poi5’ -ctf -aco 1A2_sub.sdf

would in fact yield identical results.

Let us run

sdfconf manual_test_sdf_1.sdf -aco 1A2_sub.sdf -ctf -ca ‘(49.51, 47.21, 57.36), poi’ -out manual_test_sdf_2.sdf

Now we don’t create poi5 and the csv-file, but we write all conformations with added conformation numbers, combined data and calculated distances in sdf-format to *manual_test_2.sdf*. This file is stored in appendix C.

Use as a library

After you have installed sdfconf, you may import the package to a python interpreter like ipython or to external script to help in parsing and handling data.

Internal structures

The package sdfconf includes a few modules. In general their internal functions are quite hierarchical. In following sections the modules and their most important classes are discussed.

runner

runner.py is responsible for handling of options given to sdfconf. It includes the runner class and command line UI.

runner

Runner is in essence an interface between user interface and sdfconf itself. It defines how certain options are used and in what order. A single runner instance may include a single instance of Sdffile to which all operations are made. It also stores general run information like state of verbosity or proportion options. It is also responsible for all the messages generated by verbose option.

sdf

Sdffile

Sdffile is a class that options operations on the stage of complete SDfiles.
It contains a python dictionary (._dictomoles), whose keys are common names of molecules in the file. Each keys corresponds to another dictionary which contains all conformations of molecules with the same name. Each conformation has a distinct conformation number (distinct for each molecule name). Each conformation is represented by a single instance of Sdfmole class.
Sdffile also includes a list (._orderlist) of moleculename-conformation number pairs and the order of that list describes in which order molecules are in a file.

Sdfmole

Sdfmole is responsible for storing data of a single conformation. It stores in it's own structures data included in standard V2000 Molfile. That includes header block, counts line, atom block, bond block and properties block. All molecular data is initially stored in "dumb" format, i.e. it's just stored as list of strings.
It also has a list of metanames that are associated to that conformation. Further it includes a dictionary that includes said metanames and an Sdfmeta instance for each name.
Sdfmole class also contains methods that are used to manipulate single conformations. It also harbours the dreaded molelogic (and further tabiter) method that is mostly responsible for performing the magic of metastatements.
Sdfmole along with Mol2Mol has the method atomsGenerator that is used in all structure based queries.

Sdfmeta

A single instance of Sdfmeta class includes data associated with a single datablock of a SDfile. So every datablock in a SDfile is stored in a single instance of Sdfmeta. Sdfmeta stores

information of it’s datastructure, datatype, possible delimiters, etc. As a class it also contains methods for handling operations within and between Sdfmeta.

mol2

Mol2File

Mol2File is an analogous to Sdffile, although it has only a few methods. It includes a list of Mol2Mol instances.

Mol2Mol

Mol2Mol is analogouts to Sdfmole, but it has only few methods and is much less organized.
Mol2Mol along with Sdfmole has the method atomsGenerator that is used in structure based queries.

functions

Includes various functions used in other modules.

Findable

Findable

Findable is a helping class that includes a set of coordinates and can be used to if some of them are within range of some other point. Can be used for example in checking overlapping of two molecules. Approximately at least 10 times faster than brute force point to point comparison with datasets of size >100.

Appendix

Following sections contain additional data, like files used in examples.

Appendix A

manual_test_sdf_1.sdf

Example SDfile. File is from docking software ShaEP and run from similar .mol2-files. Names were preapplied.

```
NDEA{[2]}
  1019                 3D

 17 16  0  0  0  0  0  0  0  0999 V2000
   48.0099   43.0534   54.4614 N   0  0  0  0  0  0  0  0  0  0  0  0
   46.8856   42.7739   54.8941 O   0  0  0  0  0  0  0  0  0  0  0  0
   48.9186   42.2316   54.9522 N   0  0  0  0  0  0  0  0  0  0  0  0
   48.4113   41.4768   56.1171 C   0  0  0  0  0  0  0  0  0  0  0  0
   49.4532   40.5158   56.6909 C   0  0  0  0  0  0  0  0  0  0  0  0
   50.1858   42.9195   55.2888 C   0  0  0  0  0  0  0  0  0  0  0  0
   50.0184   43.9293   56.4214 C   0  0  0  0  0  0  0  0  0  0  0  0
   48.1482   42.1618   56.9303 H   0  0  0  0  0  0  0  0  0  0  0  0
   47.4972   40.9220   55.8645 H   0  0  0  0  0  0  0  0  0  0  0  0
   49.0633   40.0422   57.5980 H   0  0  0  0  0  0  0  0  0  0  0  0
   49.7066   39.7187   55.9846 H   0  0  0  0  0  0  0  0  0  0  0  0
   50.3726   41.0473   56.9588 H   0  0  0  0  0  0  0  0  0  0  0  0
   50.6192   43.4094   54.4066 H   0  0  0  0  0  0  0  0  0  0  0  0
   50.9339   42.1933   55.6372 H   0  0  0  0  0  0  0  0  0  0  0  0
   50.9981   44.3157   56.7215 H   0  0  0  0  0  0  0  0  0  0  0  0
   49.4068   44.7865   56.1268 H   0  0  0  0  0  0  0  0  0  0  0  0
   49.5606   43.4624   57.3000 H   0  0  0  0  0  0  0  0  0  0  0  0
  1  2  2  0  0  0  0
  1  3  1  0  0  0  0
  3  4  1  0  0  0  0
  3  6  1  0  0  0  0
  4  5  1  0  0  0  0
  4  8  1  0  0  0  0
  4  9  1  0  0  0  0
  5 10  1  0  0  0  0
  5 11  1  0  0  0  0
  5 12  1  0  0  0  0
  6  7  1  0  0  0  0
  6 13  1  0  0  0  0
  6 14  1  0  0  0  0
  7 15  1  0  0  0  0
  7 16  1  0  0  0  0
  7 17  1  0  0  0  0
M  END
>  <Similarity_ESP>
0.19160430096807685

>  <Similarity_best>
0.26001664182721468

>  <Similarity_hit>
tasku_input1-agonist

>  <Similarity_shape>
0.32842898268635251

$$$$
NDEA{[1]}
  1019                 3D

 17 16  0  0  0  0  0  0  0  0999 V2000
   48.4460   41.8827   56.6634 N   0  0  0  0  0  0  0  0  0  0  0  0
   49.1576   40.8780   56.5461 O   0  0  0  0  0  0  0  0  0  0  0  0
   48.3022   42.4932   55.5023 N   0  0  0  0  0  0  0  0  0  0  0  0
   47.5541   41.6677   54.5313 C   0  0  0  0  0  0  0  0  0  0  0  0
   46.2727   41.0818   55.1256 C   0  0  0  0  0  0  0  0  0  0  0  0
   49.5936   42.9331   54.9266 C   0  0  0  0  0  0  0  0  0  0  0  0
   50.3365   43.9071   55.8375 C   0  0  0  0  0  0  0  0  0  0  0  0
   47.2426   42.2749   53.6745 H   0  0  0  0  0  0  0  0  0  0  0  0
   48.1798   40.8575   54.1323 H   0  0  0  0  0  0  0  0  0  0  0  0
   45.7122   40.5493   54.3500 H   0  0  0  0  0  0  0  0  0  0  0  0
   46.4838   40.3688   55.9292 H   0  0  0  0  0  0  0  0  0  0  0  0
   45.6269   41.8719   55.5234 H   0  0  0  0  0  0  0  0  0  0  0  0
   49.4552   43.3765   53.9315 H   0  0  0  0  0  0  0  0  0  0  0  0
   50.2640   42.0726   54.7906 H   0  0  0  0  0  0  0  0  0  0  0  0
   51.3361   44.1024   55.4346 H   0  0  0  0  0  0  0  0  0  0  0  0
   49.8255   44.8702   55.9207 H   0  0  0  0  0  0  0  0  0  0  0  0
   50.4577   43.4907   56.8430 H   0  0  0  0  0  0  0  0  0  0  0  0
  1  2  2  0  0  0  0
  1  3  1  0  0  0  0
  3  4  1  0  0  0  0
  3  6  1  0  0  0  0
  4  5  1  0  0  0  0
  4  8  1  0  0  0  0
  4  9  1  0  0  0  0
  5 10  1  0  0  0  0
  5 11  1  0  0  0  0
  5 12  1  0  0  0  0
  6  7  1  0  0  0  0
  6 13  1  0  0  0  0
  6 14  1  0  0  0  0
  7 15  1  0  0  0  0
  7 16  1  0  0  0  0
  7 17  1  0  0  0  0
M  END
>  <Similarity_ESP>
0.17809401562061908

>  <Similarity_best>
0.26931703790776473

>  <Similarity_hit>
tasku_input1-agonist

>  <Similarity_shape>
0.36054006019491042

$$$$
phenytoin{[4]}
  1019                 3D

 30 32  0  0  0  0  0  0  0  0999 V2000
   45.8422   40.7406   57.3034 C   0  0  0  0  0  0  0  0  0  0  0  0
   46.2322   39.6575   58.0274 N   0  0  0  0  0  0  0  0  0  0  0  0
   46.4289   40.5890   55.8997 C   0  0  0  0  0  0  0  0  0  0  0  0
   46.9784   38.8086   57.2814 C   0  0  0  0  0  0  0  0  0  0  0  0
   47.1234   39.2797   56.0447 N   0  5  0  0  0  0  0  0  0  0  0  0
   45.1444   41.6614   57.7061 O   0  0  0  0  0  0  0  0  0  0  0  0
   47.4369   37.7573   57.7165 O   0  0  0  0  0  0  0  0  0  0  0  0
   45.2859   40.5263   54.8744 C   0  0  0  0  0  0  0  0  0  0  0  0
   47.5104   41.6156   55.5369 C   0  0  0  0  0  0  0  0  0  0  0  0
   43.9268   40.6233   55.2220 C   0  0  0  0  0  0  0  0  0  0  0  0
   45.6000   40.3793   53.5079 C   0  0  0  0  0  0  0  0  0  0  0  0
   47.1539   42.9658   55.3374 C   0  0  0  0  0  0  0  0  0  0  0  0
   48.8624   41.2688   55.3747 C   0  0  0  0  0  0  0  0  0  0  0  0
   44.6001   40.3225   52.5343 C   0  0  0  0  0  0  0  0  0  0  0  0
   48.1104   43.9276   55.0045 C   0  0  0  0  0  0  0  0  0  0  0  0
   49.8191   42.2330   55.0413 C   0  0  0  0  0  0  0  0  0  0  0  0
   42.9265   40.5663   54.2465 C   0  0  0  0  0  0  0  0  0  0  0  0
   43.2625   40.4150   52.9044 C   0  0  0  0  0  0  0  0  0  0  0  0
   49.4441   43.5609   54.8583 C   0  0  0  0  0  0  0  0  0  0  0  0
   45.9870   39.4988   58.9955 H   0  0  0  0  0  0  0  0  0  0  0  0
   43.6173   40.7447   56.2567 H   0  0  0  0  0  0  0  0  0  0  0  0
   46.6381   40.3084   53.1892 H   0  0  0  0  0  0  0  0  0  0  0  0
   46.1168   43.2788   55.4379 H   0  0  0  0  0  0  0  0  0  0  0  0
   49.2015   40.2447   55.5002 H   0  0  0  0  0  0  0  0  0  0  0  0
   44.8687   40.2074   51.4884 H   0  0  0  0  0  0  0  0  0  0  0  0
   47.8105   44.9611   54.8578 H   0  0  0  0  0  0  0  0  0  0  0  0
   50.8591   41.9424   54.9228 H   0  0  0  0  0  0  0  0  0  0  0  0
   41.8829   40.6416   54.5391 H   0  0  0  0  0  0  0  0  0  0  0  0
   42.4835   40.3716   52.1491 H   0  0  0  0  0  0  0  0  0  0  0  0
   50.1893   44.3067   54.5986 H   0  0  0  0  0  0  0  0  0  0  0  0
  1  2  1  0  0  0  0
  1  3  1  0  0  0  0
  1  6  2  0  0  0  0
  2  4  1  0  0  0  0
  2 20  1  0  0  0  0
  3  5  1  0  0  0  0
  3  8  1  0  0  0  0
  3  9  1  0  0  0  0
  4  5  1  0  0  0  0
  4  7  2  0  0  0  0
  8 10  2  0  0  0  0
  8 11  1  0  0  0  0
  9 12  2  0  0  0  0
  9 13  1  0  0  0  0
 10 17  1  0  0  0  0
 10 21  1  0  0  0  0
```

```
 11 14  2  0  0  0  0
 11 22  1  0  0  0  0
 12 15  1  0  0  0  0
 12 23  1  0  0  0  0
 13 16  2  0  0  0  0
 13 24  1  0  0  0  0
 14 18  1  0  0  0  0
 14 25  1  0  0  0  0
 15 19  2  0  0  0  0
 15 26  1  0  0  0  0
 16 19  1  0  0  0  0
 16 27  1  0  0  0  0
 17 18  2  0  0  0  0
 17 28  1  0  0  0  0
 18 29  1  0  0  0  0
 19 30  1  0  0  0  0
M  CHG  1   5  -1
M  END
>  <Similarity_ESP>
0.44650263249175864

>  <Similarity_best>
0.48728642262229038

>  <Similarity_hit>
tasku_input1-agonist

>  <Similarity_shape>
0.52807021275282207

$$$$
phenytoin{[3]}
  1019                  3D

 30 32  0  0  0  0  0  0  0  0999 V2000
   46.5951   39.8584   57.9990 C   0  0  0  0  0  0  0  0  0  0  0  0
   46.9755   38.6281   58.4358 N   0  0  0  0  0  0  0  0  0  0  0  0
   46.9500   39.9478   56.5145 C   0  0  0  0  0  0  0  0  0  0  0  0
   47.5135   37.8936   57.4331 C   0  0  0  0  0  0  0  0  0  0  0  0
   47.5218   38.5899   56.2984 N   0  5  0  0  0  0  0  0  0  0  0  0
   46.0613   40.7278   58.6743 O   0  0  0  0  0  0  0  0  0  0  0  0
   47.9221   36.7468   57.5846 O   0  0  0  0  0  0  0  0  0  0  0  0
   45.6681   40.1717   55.6972 C   0  0  0  0  0  0  0  0  0  0  0  0
   48.0648   40.9465   56.1755 C   0  0  0  0  0  0  0  0  0  0  0  0
   44.8724   39.1239   55.2010 C   0  0  0  0  0  0  0  0  0  0  0  0
   45.2456   41.4910   55.4353 C   0  0  0  0  0  0  0  0  0  0  0  0
   48.1918   41.4316   54.8570 C   0  0  0  0  0  0  0  0  0  0  0  0
   48.9938   41.3993   57.1276 C   0  0  0  0  0  0  0  0  0  0  0  0
   44.0852   41.7505   54.7024 C   0  0  0  0  0  0  0  0  0  0  0  0
   49.1948   42.3399   54.5111 C   0  0  0  0  0  0  0  0  0  0  0  0
   49.9978   42.3087   56.7798 C   0  0  0  0  0  0  0  0  0  0  0  0
   43.7107   39.3847   54.4675 C   0  0  0  0  0  0  0  0  0  0  0  0
   43.3180   40.6963   54.2175 C   0  0  0  0  0  0  0  0  0  0  0  0
   50.0971   42.7799   55.4738 C   0  0  0  0  0  0  0  0  0  0  0  0
   46.8608   38.2977   59.3846 H   0  0  0  0  0  0  0  0  0  0  0  0
   45.1382   38.0843   55.3736 H   0  0  0  0  0  0  0  0  0  0  0  0
   45.8239   42.3349   55.8056 H   0  0  0  0  0  0  0  0  0  0  0  0
   47.5043   41.0984   54.0825 H   0  0  0  0  0  0  0  0  0  0  0  0
   48.9606   41.0544   58.1570 H   0  0  0  0  0  0  0  0  0  0  0  0
   43.7823   42.7762   54.5132 H   0  0  0  0  0  0  0  0  0  0  0  0
   49.2704   42.6996   53.4892 H   0  0  0  0  0  0  0  0  0  0  0  0
   50.7042   42.6464   57.5329 H   0  0  0  0  0  0  0  0  0  0  0  0
   43.1128   38.5589   54.0926 H   0  0  0  0  0  0  0  0  0  0  0  0
   42.4153   40.8958   53.6480 H   0  0  0  0  0  0  0  0  0  0  0  0
   50.8784   43.4851   55.2070 H   0  0  0  0  0  0  0  0  0  0  0  0
  1  2  1  0  0  0  0
  1  3  1  0  0  0  0
  1  6  2  0  0  0  0
  2  4  1  0  0  0  0
  2 20  1  0  0  0  0
  3  5  1  0  0  0  0
  3  8  1  0  0  0  0
  3  9  1  0  0  0  0
  4  5  1  0  0  0  0
  4  7  2  0  0  0  0
  8 10  2  0  0  0  0
  8 11  1  0  0  0  0
  9 12  2  0  0  0  0
  9 13  1  0  0  0  0
 10 17  1  0  0  0  0
 10 21  1  0  0  0  0
 11 14  2  0  0  0  0
 11 22  1  0  0  0  0
 12 15  1  0  0  0  0
 12 23  1  0  0  0  0
 13 16  2  0  0  0  0
 13 24  1  0  0  0  0
 14 18  1  0  0  0  0
 14 25  1  0  0  0  0
 15 19  2  0  0  0  0
 15 26  1  0  0  0  0
 16 19  1  0  0  0  0
 16 27  1  0  0  0  0
 17 18  2  0  0  0  0
 17 28  1  0  0  0  0
 18 29  1  0  0  0  0
 19 30  1  0  0  0  0
M  CHG  1   5  -1
M  END
>  <Similarity_ESP>
0.41099668007610624

>  <Similarity_best>
0.49293707562267014

>  <Similarity_hit>
tasku_input1-agonist

>  <Similarity_shape>
0.57487747116923404

$$$$
phenytoin{[1]}
  1019                  3D

 31 33  0  0  0  0  0  0  0  0999 V2000
   47.7246   38.8944   55.3811 C   0  0  0  0  0  0  0  0  0  0  0  0
   47.8106   37.9793   56.3985 N   0  0  0  0  0  0  0  0  0  0  0  0
   47.0837   40.2003   55.9142 C   0  0  0  0  0  0  0  0  0  0  0  0
   47.3218   38.4907   57.5689 C   0  0  0  0  0  0  0  0  0  0  0  0
   46.8965   39.7846   57.3034 N   0  0  0  0  0  0  0  0  0  0  0  0
   48.1062   38.7034   54.2327 O   0  0  0  0  0  0  0  0  0  0  0  0
   47.2674   37.9214   58.6489 O   0  0  0  0  0  0  0  0  0  0  0  0
   48.0907   41.3460   55.7542 C   0  0  0  0  0  0  0  0  0  0  0  0
   45.7067   40.5231   55.3244 C   0  0  0  0  0  0  0  0  0  0  0  0
   48.8691   41.8215   56.8234 C   0  0  0  0  0  0  0  0  0  0  0  0
   48.2674   41.9489   54.4934 C   0  0  0  0  0  0  0  0  0  0  0  0
   45.4201   40.1419   53.9976 C   0  0  0  0  0  0  0  0  0  0  0  0
   44.7100   41.2094   56.0368 C   0  0  0  0  0  0  0  0  0  0  0  0
   49.1788   42.9913   54.3123 C   0  0  0  0  0  0  0  0  0  0  0  0
   44.1827   40.4226   53.4144 C   0  0  0  0  0  0  0  0  0  0  0  0
   43.4714   41.4896   55.4511 C   0  0  0  0  0  0  0  0  0  0  0  0
   49.7809   42.8651   56.6400 C   0  0  0  0  0  0  0  0  0  0  0  0
   49.9347   43.4501   55.3863 C   0  0  0  0  0  0  0  0  0  0  0  0
   43.2073   41.0955   54.1426 C   0  0  0  0  0  0  0  0  0  0  0  0
   48.1946   37.0484   56.2992 H   0  0  0  0  0  0  0  0  0  0  0  0
   46.4813   40.3753   58.0163 H   0  0  0  0  0  0  0  0  0  0  0  0
   48.7845   41.3913   57.8177 H   0  0  0  0  0  0  0  0  0  0  0  0
   47.6932   41.6079   53.6344 H   0  0  0  0  0  0  0  0  0  0  0  0
   46.1656   39.6183   53.4026 H   0  0  0  0  0  0  0  0  0  0  0  0
   44.8696   41.5437   57.0577 H   0  0  0  0  0  0  0  0  0  0  0  0
   49.2979   43.4426   53.3313 H   0  0  0  0  0  0  0  0  0  0  0  0
   43.9833   40.1162   52.3916 H   0  0  0  0  0  0  0  0  0  0  0  0
   42.7126   42.0194   56.0205 H   0  0  0  0  0  0  0  0  0  0  0  0
   50.3726   43.2196   57.4795 H   0  0  0  0  0  0  0  0  0  0  0  0
   50.6443   44.2604   55.2467 H   0  0  0  0  0  0  0  0  0  0  0  0
   42.2444   41.3153   53.6911 H   0  0  0  0  0  0  0  0  0  0  0  0
  1  2  1  0  0  0  0
  1  3  1  0  0  0  0
  1  6  2  0  0  0  0
  2  4  1  0  0  0  0
  2 20  1  0  0  0  0
  3  5  1  0  0  0  0
  3  8  1  0  0  0  0
  3  9  1  0  0  0  0
  4  5  1  0  0  0  0
  4  7  2  0  0  0  0
  5 21  1  0  0  0  0
  8 10  2  0  0  0  0
  8 11  1  0  0  0  0
  9 12  2  0  0  0  0
  9 13  1  0  0  0  0
 10 17  1  0  0  0  0
 10 22  1  0  0  0  0
 11 14  2  0  0  0  0
 11 23  1  0  0  0  0
 12 15  1  0  0  0  0
 12 24  1  0  0  0  0
 13 16  2  0  0  0  0
 13 25  1  0  0  0  0
 14 18  1  0  0  0  0
 14 26  1  0  0  0  0
 15 19  2  0  0  0  0
 15 27  1  0  0  0  0
 16 19  1  0  0  0  0
 16 28  1  0  0  0  0
 17 18  2  0  0  0  0
 17 29  1  0  0  0  0
 18 30  1  0  0  0  0
 19 31  1  0  0  0  0
M  END
>  <Similarity_ESP>
0.45409357855608479

>  <Similarity_best>
0.49832632860591874

>  <Similarity_hit>
```

```
tasku_input1-agonist

>  <Similarity_shape>
0.5425590786557527

$$$$
phenytoin{[2]}
  1019                  3D

 30 32  0  0  0  0  0  0  0  0999 V2000
   47.8991   39.0125   55.1113 C   0  0  0  0  0  0  0  0  0  0  0  0
   48.0895   38.0065   55.9747 N   0  5  0  0  0  0  0  0  0  0  0  0
   47.2628   40.2439   55.7863 C   0  0  0  0  0  0  0  0  0  0  0  0
   47.6794   38.3984   57.1718 C   0  0  0  0  0  0  0  0  0  0  0  0
   47.2026   39.6756   57.1062 N   0  0  0  0  0  0  0  0  0  0  0  0
   48.1935   38.9869   53.9184 O   0  0  0  0  0  0  0  0  0  0  0  0
   47.7058   37.7432   58.2084 O   0  0  0  0  0  0  0  0  0  0  0  0
   48.2170   41.4405   55.6738 C   0  0  0  0  0  0  0  0  0  0  0  0
   45.8364   40.5727   55.3311 C   0  0  0  0  0  0  0  0  0  0  0  0
   49.0440   41.8480   56.7347 C   0  0  0  0  0  0  0  0  0  0  0  0
   48.2883   42.1661   54.4688 C   0  0  0  0  0  0  0  0  0  0  0  0
   45.4711   40.3383   53.9896 C   0  0  0  0  0  0  0  0  0  0  0  0
   44.8663   41.1197   56.1862 C   0  0  0  0  0  0  0  0  0  0  0  0
   49.1455   43.2603   54.3337 C   0  0  0  0  0  0  0  0  0  0  0  0
   44.1851   40.6269   53.5281 C   0  0  0  0  0  0  0  0  0  0  0  0
   43.5790   41.4083   55.7226 C   0  0  0  0  0  0  0  0  0  0  0  0
   49.9015   42.9434   56.5978 C   0  0  0  0  0  0  0  0  0  0  0  0
   49.9514   43.6495   55.3991 C   0  0  0  0  0  0  0  0  0  0  0  0
   43.2382   41.1609   54.3958 C   0  0  0  0  0  0  0  0  0  0  0  0
   46.8285   40.1579   57.9158 H   0  0  0  0  0  0  0  0  0  0  0  0
   49.0395   41.3208   57.6849 H   0  0  0  0  0  0  0  0  0  0  0  0
   47.6730   41.8800   53.6184 H   0  0  0  0  0  0  0  0  0  0  0  0
   46.1932   39.9251   53.2887 H   0  0  0  0  0  0  0  0  0  0  0  0
   45.0883   41.3364   57.2271 H   0  0  0  0  0  0  0  0  0  0  0  0
   49.1837   43.8056   53.3951 H   0  0  0  0  0  0  0  0  0  0  0  0
   43.9257   40.4353   52.4908 H   0  0  0  0  0  0  0  0  0  0  0  0
   42.8431   41.8299   56.4017 H   0  0  0  0  0  0  0  0  0  0  0  0
   50.5328   43.2425   57.4298 H   0  0  0  0  0  0  0  0  0  0  0  0
   50.6192   44.4995   55.2950 H   0  0  0  0  0  0  0  0  0  0  0  0
   42.2377   41.3870   54.0392 H   0  0  0  0  0  0  0  0  0  0  0  0
  1  2  1  0  0  0  0
  1  3  1  0  0  0  0
  1  6  2  0  0  0  0
  2  4  1  0  0  0  0
  3  5  1  0  0  0  0
  3  8  1  0  0  0  0
  3  9  1  0  0  0  0
  4  5  1  0  0  0  0
  4  7  2  0  0  0  0
  5 20  1  0  0  0  0
  8 10  2  0  0  0  0
  8 11  1  0  0  0  0
  9 12  2  0  0  0  0
  9 13  1  0  0  0  0
 10 17  1  0  0  0  0
 10 21  1  0  0  0  0
 11 14  2  0  0  0  0
 11 22  1  0  0  0  0
 12 15  1  0  0  0  0
 12 23  1  0  0  0  0
 13 16  2  0  0  0  0
 13 24  1  0  0  0  0
 14 18  1  0  0  0  0
 14 25  1  0  0  0  0
 15 19  2  0  0  0  0
 15 26  1  0  0  0  0
 16 19  1  0  0  0  0
 16 27  1  0  0  0  0
 17 18  2  0  0  0  0
 17 28  1  0  0  0  0
 18 29  1  0  0  0  0
 19 30  1  0  0  0  0
M  CHG  1   2  -1
M  END
>  <Similarity_ESP>
0.46565058557634675

>  <Similarity_best>
0.50484238357349409

>  <Similarity_hit>
tasku_input1-agonist

>  <Similarity_shape>
0.54403418157064143

$$$$
```

Appendix B

1A2_sub.sdf

Example file including some basic information on 2 molecules. This is a subset of actual file used as a starting point in a study.

```
NDEA
  MOE2008              3D

  7  6  0  0  0  0  0  0  0  0999 V2000
   -1.4200    2.1290    0.5220 N   0  0  0  0  0  0  0  0  0  0  0  0
   -2.3850    1.7790    1.2100 O   0  0  0  0  0  0  0  0  0  0  0  0
   -1.3770    3.4350    0.4020 N   0  0  0  0  0  0  0  0  0  0  0  0
   -0.0280    3.8820    0.0740 C   0  0  0  0  0  0  0  0  0  0  0  0
    0.4090    3.6260   -1.3550 C   0  0  0  0  0  0  0  0  0  0  0  0
   -2.2950    3.9590   -0.6050 C   0  0  0  0  0  0  0  0  0  0  0  0
   -3.7110    4.0650   -0.0840 C   0  0  0  0  0  0  0  0  0  0  0  0
  1  2  2  0  0  0  0
  1  3  1  0  0  0  0
  3  4  1  0  0  0  0
  3  6  1  0  0  0  0
  4  5  1  0  0  0  0
  6  7  1  0  0  0  0
M  END
>  <ID>
NDEA

>  <PRIMARY_SOM>
4 6

>  <Citation>
Bellec et al., Carcinogenesis, 1996, 17, 2029-2034

$$$$
phenytoin
  MOE2008              3D

 19 21  0  0  0  0  0  0  0  0999 V2000
   -0.0570    1.5580    0.1280 C   0  0  0  0  0  0  0  0  0  0  0  0
   -0.2250    0.2250    0.2410 N   0  0  0  0  0  0  0  0  0  0  0  0
    1.4370    1.7730   -0.0020 C   0  0  0  0  0  0  0  0  0  0  0  0
    0.9440   -0.4240    0.2000 C   0  0  0  0  0  0  0  0  0  0  0  0
    1.9900    0.4090    0.0580 N   0  0  0  0  0  0  0  0  0  0  0  0
   -0.9220    2.4070    0.1260 O   0  0  0  0  0  0  0  0  0  0  0  0
    1.0490   -1.6320    0.2820 O   0  0  0  0  0  0  0  0  0  0  0  0
    1.7660    2.4210   -1.3220 C   0  0  0  0  0  0  0  0  0  0  0  0
    1.9550    2.6060    1.1420 C   0  0  0  0  0  0  0  0  0  0  0  0
    2.7410    1.8750   -2.1360 C   0  0  0  0  0  0  0  0  0  0  0  0
    1.0900    3.5590   -1.7210 C   0  0  0  0  0  0  0  0  0  0  0  0
    3.0640    2.1880    1.8550 C   0  0  0  0  0  0  0  0  0  0  0  0
    1.3250    3.7900    1.4760 C   0  0  0  0  0  0  0  0  0  0  0  0
    1.3920    4.1530   -2.9320 C   0  0  0  0  0  0  0  0  0  0  0  0
    3.5380    2.9510    2.9050 C   0  0  0  0  0  0  0  0  0  0  0  0
    1.8000    4.5530    2.5260 C   0  0  0  0  0  0  0  0  0  0  0  0
    3.0430    2.4700   -3.3470 C   0  0  0  0  0  0  0  0  0  0  0  0
    2.3710    3.6110   -3.7440 C   0  0  0  0  0  0  0  0  0  0  0  0
    2.9050    4.1330    3.2420 C   0  0  0  0  0  0  0  0  0  0  0  0
  1  2  1  0  0  0  0
  1  3  1  0  0  0  0
  1  6  2  0  0  0  0
  2  4  1  0  0  0  0
  3  5  1  0  0  0  0
  3  8  1  0  0  0  0
  3  9  1  0  0  0  0
  4  5  1  0  0  0  0
  4  7  2  0  0  0  0
  8 10  2  0  0  0  0
  8 11  1  0  0  0  0
  9 12  2  0  0  0  0
  9 13  1  0  0  0  0
 10 17  1  0  0  0  0
 11 14  2  0  0  0  0
 12 15  1  0  0  0  0
 13 16  2  0  0  0  0
 14 18  1  0  0  0  0
 15 19  2  0  0  0  0
 16 19  1  0  0  0  0
 17 18  2  0  0  0  0
M  END
>  <ID>
phenytoin

>  <PRIMARY_SOM>
14 15 16 17 18 19

>  <Citation>
Komatsu et al.,Drug Metab. Disp.,28,1361,2000

$$$$
```

Appendix C

File written by example *closestatoms, allcombine, getcsv, conftometa, makenewmeta.*

```
NDEA{[2]}
1019                    3D

 17 16  0  0  0  0  0  0  0  0999 V2000
   48.0099   43.0534   54.4614 N   0  0  0  0  0  0  0  0  0  0  0  0
   46.8856   42.7739   54.8941 O   0  0  0  0  0  0  0  0  0  0  0  0
   48.9186   42.2316   54.9522 N   0  0  0  0  0  0  0  0  0  0  0  0
   48.4113   41.4768   56.1171 C   0  0  0  0  0  0  0  0  0  0  0  0
   49.4532   40.5158   56.6909 C   0  0  0  0  0  0  0  0  0  0  0  0
   50.1858   42.9195   55.2888 C   0  0  0  0  0  0  0  0  0  0  0  0
   50.0184   43.9293   56.4214 C   0  0  0  0  0  0  0  0  0  0  0  0
   48.1482   42.1618   56.9303 H   0  0  0  0  0  0  0  0  0  0  0  0
   47.4972   40.9220   55.8645 H   0  0  0  0  0  0  0  0  0  0  0  0
   49.0633   40.0422   57.5980 H   0  0  0  0  0  0  0  0  0  0  0  0
   49.7066   39.7187   55.9846 H   0  0  0  0  0  0  0  0  0  0  0  0
   50.3726   41.0473   56.9588 H   0  0  0  0  0  0  0  0  0  0  0  0
   50.6192   43.4094   54.4066 H   0  0  0  0  0  0  0  0  0  0  0  0
   50.9339   42.1933   55.6372 H   0  0  0  0  0  0  0  0  0  0  0  0
   50.9981   44.3157   56.7215 H   0  0  0  0  0  0  0  0  0  0  0  0
   49.4068   44.7865   56.1268 H   0  0  0  0  0  0  0  0  0  0  0  0
   49.5606   43.4624   57.3000 H   0  0  0  0  0  0  0  0  0  0  0  0
  1  2  2  0  0  0  0
  1  3  1  0  0  0  0
  3  4  1  0  0  0  0
  3  6  1  0  0  0  0
  4  5  1  0  0  0  0
  4  8  1  0  0  0  0
  4  9  1  0  0  0  0
  5 10  1  0  0  0  0
  5 11  1  0  0  0  0
  5 12  1  0  0  0  0
  6  7  1  0  0  0  0
  6 13  1  0  0  0  0
  6 14  1  0  0  0  0
  7 15  1  0  0  0  0
  7 16  1  0  0  0  0
  7 17  1  0  0  0  0
M  END
>  <Similarity_ESP>
0.19160430096807685

>  <Similarity_best>
0.26001664182721468

>  <Similarity_hit>
tasku_input1-agonist

>  <Similarity_shape>
0.32842898268635251

>  <confnum>
2

>  <ID>
NDEA

>  <PRIMARY_SOM>
4 6

>  <Citation>
Bellec et al., Carcinogenesis, 1996, 17, 2029-2034

>  <poi>
7:3.45, 6:4.812, 1:5.2848, 3:5.5616, 2:5.7138, 4:5.9684, 5:6.7278

$$$$
NDEA{[1]}
1019                    3D

 17 16  0  0  0  0  0  0  0  0999 V2000
   48.4460   41.8827   56.6634 N   0  0  0  0  0  0  0  0  0  0  0  0
   49.1576   40.8780   56.5461 O   0  0  0  0  0  0  0  0  0  0  0  0
   48.3022   42.4932   55.5023 N   0  0  0  0  0  0  0  0  0  0  0  0
   47.5541   41.6677   54.5313 C   0  0  0  0  0  0  0  0  0  0  0  0
   46.2727   41.0818   55.1256 C   0  0  0  0  0  0  0  0  0  0  0  0
   49.5936   42.9331   54.9266 C   0  0  0  0  0  0  0  0  0  0  0  0
   50.3365   43.9071   55.8375 C   0  0  0  0  0  0  0  0  0  0  0  0
   47.2426   42.2749   53.6745 H   0  0  0  0  0  0  0  0  0  0  0  0
   48.1798   40.8575   54.1323 H   0  0  0  0  0  0  0  0  0  0  0  0
   45.7122   40.5493   54.3500 H   0  0  0  0  0  0  0  0  0  0  0  0
   46.4838   40.3688   55.9292 H   0  0  0  0  0  0  0  0  0  0  0  0
   45.6269   41.8719   55.5234 H   0  0  0  0  0  0  0  0  0  0  0  0
   49.4552   43.3765   53.9315 H   0  0  0  0  0  0  0  0  0  0  0  0
   50.2640   42.0726   54.7906 H   0  0  0  0  0  0  0  0  0  0  0  0
   51.3361   44.1024   55.4346 H   0  0  0  0  0  0  0  0  0  0  0  0
   49.8255   44.8702   55.9207 H   0  0  0  0  0  0  0  0  0  0  0  0
   50.4577   43.4907   56.8430 H   0  0  0  0  0  0  0  0  0  0  0  0
  1  2  2  0  0  0  0
  1  3  1  0  0  0  0
  3  4  1  0  0  0  0
  3  6  1  0  0  0  0
  4  5  1  0  0  0  0
  4  8  1  0  0  0  0
  4  9  1  0  0  0  0
  5 10  1  0  0  0  0
  5 11  1  0  0  0  0
  5 12  1  0  0  0  0
  6  7  1  0  0  0  0
  6 13  1  0  0  0  0
  6 14  1  0  0  0  0
  7 15  1  0  0  0  0
  7 16  1  0  0  0  0
  7 17  1  0  0  0  0
M  END
>  <Similarity_ESP>
0.17809401562061908

>  <Similarity_best>
0.26931703790776473

>  <Similarity_hit>
tasku_input1-agonist

>  <Similarity_shape>
0.36054006019491042

>  <confnum>
1

>  <ID>
NDEA

>  <PRIMARY_SOM>
4 6

>  <Citation>
Bellec et al., Carcinogenesis, 1996, 17, 2029-2034

>  <poi>
7:3.7296, 6:4.9214, 3:5.2113, 1:5.477, 2:6.3938, 4:6.5226, 5:7.282

$$$$
phenytoin{[4]}
1019                    3D

 30 32  0  0  0  0  0  0  0  0999 V2000
   45.8422   40.7406   57.3034 C   0  0  0  0  0  0  0  0  0  0  0  0
   46.2322   39.6575   58.0274 N   0  0  0  0  0  0  0  0  0  0  0  0
   46.4289   40.5890   55.8997 C   0  0  0  0  0  0  0  0  0  0  0  0
   46.9784   38.8086   57.2814 C   0  0  0  0  0  0  0  0  0  0  0  0
   47.1234   39.2797   56.0447 N   0  5  0  0  0  0  0  0  0  0  0  0
   45.1444   41.6614   57.7061 O   0  0  0  0  0  0  0  0  0  0  0  0
   47.4369   37.7573   57.7165 O   0  0  0  0  0  0  0  0  0  0  0  0
   45.2859   40.5263   54.8744 C   0  0  0  0  0  0  0  0  0  0  0  0
   47.5104   41.6156   55.5369 C   0  0  0  0  0  0  0  0  0  0  0  0
   43.9268   40.6233   55.2220 C   0  0  0  0  0  0  0  0  0  0  0  0
   45.6000   40.3793   53.5079 C   0  0  0  0  0  0  0  0  0  0  0  0
   47.1539   42.9658   55.3374 C   0  0  0  0  0  0  0  0  0  0  0  0
   48.8624   41.2688   55.3747 C   0  0  0  0  0  0  0  0  0  0  0  0
   44.6001   40.3225   52.5343 C   0  0  0  0  0  0  0  0  0  0  0  0
   48.1104   43.9276   55.0045 C   0  0  0  0  0  0  0  0  0  0  0  0
   49.8191   42.2330   55.0413 C   0  0  0  0  0  0  0  0  0  0  0  0
   42.9265   40.5663   54.2465 C   0  0  0  0  0  0  0  0  0  0  0  0
   43.2625   40.4150   52.9044 C   0  0  0  0  0  0  0  0  0  0  0  0
   49.4441   43.5609   54.8583 C   0  0  0  0  0  0  0  0  0  0  0  0
   45.9870   39.4988   58.9955 H   0  0  0  0  0  0  0  0  0  0  0  0
   43.6173   40.7447   56.2567 H   0  0  0  0  0  0  0  0  0  0  0  0
   46.6381   40.3084   53.1892 H   0  0  0  0  0  0  0  0  0  0  0  0
   46.1168   43.2788   55.4379 H   0  0  0  0  0  0  0  0  0  0  0  0
   49.2015   40.2447   55.5002 H   0  0  0  0  0  0  0  0  0  0  0  0
   44.8687   40.2074   51.4884 H   0  0  0  0  0  0  0  0  0  0  0  0
   47.8105   44.9611   54.8578 H   0  0  0  0  0  0  0  0  0  0  0  0
   50.8591   41.9424   54.9228 H   0  0  0  0  0  0  0  0  0  0  0  0
   41.8829   40.6416   54.5391 H   0  0  0  0  0  0  0  0  0  0  0  0
   42.4835   40.3716   52.1491 H   0  0  0  0  0  0  0  0  0  0  0  0
   50.1893   44.3067   54.5986 H   0  0  0  0  0  0  0  0  0  0  0  0
  1  2  1  0  0  0  0
  1  3  1  0  0  0  0
  1  6  2  0  0  0  0
  2  4  1  0  0  0  0
  2 20  1  0  0  0  0
  3  5  1  0  0  0  0
  3  8  1  0  0  0  0
  3  9  1  0  0  0  0
```

```
  4  5  1  0  0  0  0
  4  7  2  0  0  0  0
  8 10  2  0  0  0  0
  8 11  1  0  0  0  0
  9 12  2  0  0  0  0
  9 13  1  0  0  0  0
 10 17  1  0  0  0  0
 10 21  1  0  0  0  0
 11 14  2  0  0  0  0
 11 22  1  0  0  0  0
 12 15  1  0  0  0  0
 12 23  1  0  0  0  0
 13 16  2  0  0  0  0
 13 24  1  0  0  0  0
 14 18  1  0  0  0  0
 14 25  1  0  0  0  0
 15 19  2  0  0  0  0
 15 26  1  0  0  0  0
 16 19  1  0  0  0  0
 16 27  1  0  0  0  0
 17 18  2  0  0  0  0
 17 28  1  0  0  0  0
 18 29  1  0  0  0  0
 19 30  1  0  0  0  0
M  CHG  1   5  -1
M  END
>  <Similarity_ESP>
0.44650263249175864

>  <Similarity_best>
0.48728642262229038

>  <Similarity_hit>
tasku_input1-agonist

>  <Similarity_shape>
0.52807021275282207

>  <confnum>
4

>  <ID>
phenytoin

>  <PRIMARY_SOM>
14 15 16 17 18 19

>  <Citation>
Komatsu et al.,Drug Metab. Disp.,28,1361,2000

>  <poi>
15:4.2757, 19:4.4248, 12:5.2588, 16:5.4993, 9:6.2145, 13:6.2975, 6:7.0686
, 1:7.437, 3:7.4474, 2:8.2601, 8:8.2881, 5:8.3854, 11:8.7627, 4:8.7749,
10:8.8954, 7:9.6839, 14:9.7382, 17:9.8577, 18:10.2497

$$$$
phenytoin{[3]}
1019                    3D

 30 32  0  0  0  0  0  0  0  0999 V2000
   46.5951   39.8584   57.9990 C   0  0  0  0  0  0  0  0  0  0  0  0
   46.9755   38.6281   58.4358 N   0  0  0  0  0  0  0  0  0  0  0  0
   46.9500   39.9478   56.5145 C   0  0  0  0  0  0  0  0  0  0  0  0
   47.5135   37.8936   57.4331 C   0  0  0  0  0  0  0  0  0  0  0  0
   47.5218   38.5899   56.2984 N   0  5  0  0  0  0  0  0  0  0  0  0
   46.0613   40.7278   58.6743 O   0  0  0  0  0  0  0  0  0  0  0  0
   47.9221   36.7468   57.5846 O   0  0  0  0  0  0  0  0  0  0  0  0
   45.6681   40.1717   55.6972 C   0  0  0  0  0  0  0  0  0  0  0  0
   48.0648   40.9465   56.1755 C   0  0  0  0  0  0  0  0  0  0  0  0
   44.8724   39.1239   55.2010 C   0  0  0  0  0  0  0  0  0  0  0  0
   45.2456   41.4910   55.4353 C   0  0  0  0  0  0  0  0  0  0  0  0
   48.1918   41.4316   54.8570 C   0  0  0  0  0  0  0  0  0  0  0  0
   48.9938   41.3993   57.1276 C   0  0  0  0  0  0  0  0  0  0  0  0
   44.0852   41.7505   54.7024 C   0  0  0  0  0  0  0  0  0  0  0  0
   49.1948   42.3399   54.5111 C   0  0  0  0  0  0  0  0  0  0  0  0
   49.9978   42.3087   56.7798 C   0  0  0  0  0  0  0  0  0  0  0  0
   43.7107   39.3847   54.4675 C   0  0  0  0  0  0  0  0  0  0  0  0
   43.3180   40.6963   54.2175 C   0  0  0  0  0  0  0  0  0  0  0  0
   50.0971   42.7799   55.4738 C   0  0  0  0  0  0  0  0  0  0  0  0
   46.8608   38.2977   59.3846 H   0  0  0  0  0  0  0  0  0  0  0  0
   45.1382   38.0843   55.3736 H   0  0  0  0  0  0  0  0  0  0  0  0
   45.8239   42.3349   55.8056 H   0  0  0  0  0  0  0  0  0  0  0  0
   47.5043   41.0984   54.0825 H   0  0  0  0  0  0  0  0  0  0  0  0
   48.9606   41.0544   58.1570 H   0  0  0  0  0  0  0  0  0  0  0  0
   43.7823   42.7762   54.5132 H   0  0  0  0  0  0  0  0  0  0  0  0
   49.2704   42.6996   53.4892 H   0  0  0  0  0  0  0  0  0  0  0  0
   50.7042   42.6464   57.5329 H   0  0  0  0  0  0  0  0  0  0  0  0
   43.1128   38.5589   54.0926 H   0  0  0  0  0  0  0  0  0  0  0  0
   42.4153   40.8958   53.6480 H   0  0  0  0  0  0  0  0  0  0  0  0
   50.8784   43.4851   55.2070 H   0  0  0  0  0  0  0  0  0  0  0  0
  1  2  1  0  0  0  0
  1  3  1  0  0  0  0
  1  6  2  0  0  0  0
  2  4  1  0  0  0  0
  2 20  1  0  0  0  0
  3  5  1  0  0  0  0
  3  8  1  0  0  0  0
  3  9  1  0  0  0  0
  4  5  1  0  0  0  0
  4  7  2  0  0  0  0
  8 10  2  0  0  0  0
  8 11  1  0  0  0  0
  9 12  2  0  0  0  0
  9 13  1  0  0  0  0
 10 17  1  0  0  0  0
 10 21  1  0  0  0  0
 11 14  2  0  0  0  0
 11 22  1  0  0  0  0
 12 15  1  0  0  0  0
 12 23  1  0  0  0  0
 13 16  2  0  0  0  0
 13 24  1  0  0  0  0
 14 18  1  0  0  0  0
 14 25  1  0  0  0  0
 15 19  2  0  0  0  0
 15 26  1  0  0  0  0
 16 19  1  0  0  0  0
 16 27  1  0  0  0  0
 17 18  2  0  0  0  0
 17 28  1  0  0  0  0
 18 29  1  0  0  0  0
 19 30  1  0  0  0  0
M  CHG  1   5  -1
M  END
>  <Similarity_ESP>
0.41099668007610624

>  <Similarity_best>
0.49293707562267014

>  <Similarity_hit>
tasku_input1-agonist

>  <Similarity_shape>
0.57487747116923404

>  <confnum>
3

>  <ID>
phenytoin

>  <PRIMARY_SOM>
14 15 16 17 18 19

>  <Citation>
Komatsu et al.,Drug Metab. Disp.,28,1361,2000

>  <poi>
19:4.8506, 16:4.9596, 15:5.651, 13:5.8382, 12:6.4337, 9:6.5363, 11:7.3889
, 6:7.4592, 3:7.7465, 1:7.9342, 14:8.1423, 8:8.1892, 5:8.9099, 2:9.0128,
18:9.5207, 4:9.5282, 10:9.5684, 17:10.1604, 7:10.5854

$$$$
phenytoin{[1]}
1019                    3D

 31 33  0  0  0  0  0  0  0  0999 V2000
   47.7246   38.8944   55.3811 C   0  0  0  0  0  0  0  0  0  0  0  0
   47.8106   37.9793   56.3985 N   0  0  0  0  0  0  0  0  0  0  0  0
   47.0837   40.2003   55.9142 C   0  0  0  0  0  0  0  0  0  0  0  0
   47.3218   38.4907   57.5689 C   0  0  0  0  0  0  0  0  0  0  0  0
   46.8965   39.7846   57.3034 N   0  0  0  0  0  0  0  0  0  0  0  0
   48.1062   38.7034   54.2327 O   0  0  0  0  0  0  0  0  0  0  0  0
   47.2674   37.9214   58.6489 O   0  0  0  0  0  0  0  0  0  0  0  0
   48.0907   41.3460   55.7542 C   0  0  0  0  0  0  0  0  0  0  0  0
   45.7067   40.5231   55.3244 C   0  0  0  0  0  0  0  0  0  0  0  0
   48.8691   41.8215   56.8234 C   0  0  0  0  0  0  0  0  0  0  0  0
   48.2674   41.9489   54.4934 C   0  0  0  0  0  0  0  0  0  0  0  0
   45.4201   40.1419   53.9976 C   0  0  0  0  0  0  0  0  0  0  0  0
   44.7100   41.2094   56.0368 C   0  0  0  0  0  0  0  0  0  0  0  0
   49.1788   42.9913   54.3123 C   0  0  0  0  0  0  0  0  0  0  0  0
   44.1827   40.4226   53.4144 C   0  0  0  0  0  0  0  0  0  0  0  0
   43.4714   41.4896   55.4511 C   0  0  0  0  0  0  0  0  0  0  0  0
   49.7809   42.8651   56.6400 C   0  0  0  0  0  0  0  0  0  0  0  0
   49.9347   43.4501   55.3863 C   0  0  0  0  0  0  0  0  0  0  0  0
   43.2073   41.0955   54.1426 C   0  0  0  0  0  0  0  0  0  0  0  0
   48.1946   37.0484   56.2992 H   0  0  0  0  0  0  0  0  0  0  0  0
   46.4813   40.3753   58.0163 H   0  0  0  0  0  0  0  0  0  0  0  0
   48.7845   41.3913   57.8177 H   0  0  0  0  0  0  0  0  0  0  0  0
   47.6932   41.6079   53.6344 H   0  0  0  0  0  0  0  0  0  0  0  0
   46.1656   39.6183   53.4026 H   0  0  0  0  0  0  0  0  0  0  0  0
   44.8696   41.5437   57.0577 H   0  0  0  0  0  0  0  0  0  0  0  0
   49.2979   43.4426   53.3313 H   0  0  0  0  0  0  0  0  0  0  0  0
   43.9833   40.1162   52.3916 H   0  0  0  0  0  0  0  0  0  0  0  0
   42.7126   42.0194   56.0205 H   0  0  0  0  0  0  0  0  0  0  0  0
   50.3726   43.2196   57.4795 H   0  0  0  0  0  0  0  0  0  0  0  0
   50.6443   44.2604   55.2467 H   0  0  0  0  0  0  0  0  0  0  0  0
```

```
   42.2444   41.3153   53.6911 H   0  0  0  0  0  0  0  0  0  0  0  0
  1  2  1  0  0  0  0
  1  3  1  0  0  0  0
  1  6  2  0  0  0  0
  2  4  1  0  0  0  0
  2 20  1  0  0  0  0
  3  5  1  0  0  0  0
  3  8  1  0  0  0  0
  3  9  1  0  0  0  0
  4  5  1  0  0  0  0
  4  7  2  0  0  0  0
  5 21  1  0  0  0  0
  8 10  2  0  0  0  0
  8 11  1  0  0  0  0
  9 12  2  0  0  0  0
  9 13  1  0  0  0  0
 10 17  1  0  0  0  0
 10 22  1  0  0  0  0
 11 14  2  0  0  0  0
 11 23  1  0  0  0  0
 12 15  1  0  0  0  0
 12 24  1  0  0  0  0
 13 16  2  0  0  0  0
 13 25  1  0  0  0  0
 14 18  1  0  0  0  0
 14 26  1  0  0  0  0
 15 19  2  0  0  0  0
 15 27  1  0  0  0  0
 16 19  1  0  0  0  0
 16 28  1  0  0  0  0
 17 18  2  0  0  0  0
 17 29  1  0  0  0  0
 18 30  1  0  0  0  0
 19 31  1  0  0  0  0
M  END
>  <Similarity_ESP>
0.45409357855608479

>  <Similarity_best>
0.49832632860591874

>  <Similarity_hit>
tasku_input1-agonist

>  <Similarity_shape>
0.5425590786557527

>  <confnum>
1

>  <ID>
phenytoin

>  <PRIMARY_SOM>
14 15 16 17 18 19

>  <Citation>
Komatsu et al.,Drug Metab. Disp.,28,1361,2000

>  <poi>
18:4.2676, 17:4.4125, 14:5.2149, 10:5.4529, 11:6.1189, 8:6.2434, 3:7.5573
, 13:7.7973, 5:7.8721, 9:7.9576, 16:8.5341, 1:8.7323, 12:8.8313, 4:8.9921
, 6:9.1713, 19:9.3522, 2:9.4349, 15:9.4877, 7:9.642

$$$$
phenytoin{[2]}
1019                    3D

 30 32  0  0  0  0  0  0  0  0999 V2000
   47.8991   39.0125   55.1113 C   0  0  0  0  0  0  0  0  0  0  0  0
   48.0895   38.0065   55.9747 N   0  5  0  0  0  0  0  0  0  0  0  0
   47.2628   40.2439   55.7863 C   0  0  0  0  0  0  0  0  0  0  0  0
   47.6794   38.3984   57.1718 C   0  0  0  0  0  0  0  0  0  0  0  0
   47.2026   39.6756   57.1062 N   0  0  0  0  0  0  0  0  0  0  0  0
   48.1935   38.9869   53.9184 O   0  0  0  0  0  0  0  0  0  0  0  0
   47.7058   37.7432   58.2084 O   0  0  0  0  0  0  0  0  0  0  0  0
   48.2170   41.4405   55.6738 C   0  0  0  0  0  0  0  0  0  0  0  0
   45.8364   40.5727   55.3311 C   0  0  0  0  0  0  0  0  0  0  0  0
   49.0440   41.8480   56.7347 C   0  0  0  0  0  0  0  0  0  0  0  0
   48.2883   42.1661   54.4688 C   0  0  0  0  0  0  0  0  0  0  0  0
   45.4711   40.3383   53.9896 C   0  0  0  0  0  0  0  0  0  0  0  0
   44.8663   41.1197   56.1862 C   0  0  0  0  0  0  0  0  0  0  0  0
   49.1455   43.2603   54.3337 C   0  0  0  0  0  0  0  0  0  0  0  0
   44.1851   40.6269   53.5281 C   0  0  0  0  0  0  0  0  0  0  0  0
   43.5790   41.4083   55.7226 C   0  0  0  0  0  0  0  0  0  0  0  0
   49.9015   42.9434   56.5978 C   0  0  0  0  0  0  0  0  0  0  0  0
   49.9514   43.6495   55.3991 C   0  0  0  0  0  0  0  0  0  0  0  0
   43.2382   41.1609   54.3958 C   0  0  0  0  0  0  0  0  0  0  0  0
   46.8285   40.1579   57.9158 H   0  0  0  0  0  0  0  0  0  0  0  0
   49.0395   41.3208   57.6849 H   0  0  0  0  0  0  0  0  0  0  0  0
   47.6730   41.8800   53.6184 H   0  0  0  0  0  0  0  0  0  0  0  0
   46.1932   39.9251   53.2887 H   0  0  0  0  0  0  0  0  0  0  0  0
   45.0883   41.3364   57.2271 H   0  0  0  0  0  0  0  0  0  0  0  0
   49.1837   43.8056   53.3951 H   0  0  0  0  0  0  0  0  0  0  0  0
   43.9257   40.4353   52.4908 H   0  0  0  0  0  0  0  0  0  0  0  0
   42.8431   41.8299   56.4017 H   0  0  0  0  0  0  0  0  0  0  0  0
   50.5328   43.2425   57.4298 H   0  0  0  0  0  0  0  0  0  0  0  0
   50.6192   44.4995   55.2950 H   0  0  0  0  0  0  0  0  0  0  0  0
   42.2377   41.3870   54.0392 H   0  0  0  0  0  0  0  0  0  0  0  0
  1  2  1  0  0  0  0
  1  3  1  0  0  0  0
  1  6  2  0  0  0  0
  2  4  1  0  0  0  0
  3  5  1  0  0  0  0
  3  8  1  0  0  0  0
  3  9  1  0  0  0  0
  4  5  1  0  0  0  0
  4  7  2  0  0  0  0
  5 20  1  0  0  0  0
  8 10  2  0  0  0  0
  8 11  1  0  0  0  0
  9 12  2  0  0  0  0
  9 13  1  0  0  0  0
 10 17  1  0  0  0  0
 10 21  1  0  0  0  0
 11 14  2  0  0  0  0
 11 22  1  0  0  0  0
 12 15  1  0  0  0  0
 12 23  1  0  0  0  0
 13 16  2  0  0  0  0
 13 24  1  0  0  0  0
 14 18  1  0  0  0  0
 14 25  1  0  0  0  0
 15 19  2  0  0  0  0
 15 26  1  0  0  0  0
 16 19  1  0  0  0  0
 16 27  1  0  0  0  0
 17 18  2  0  0  0  0
 17 28  1  0  0  0  0
 18 29  1  0  0  0  0
 19 30  1  0  0  0  0
M  CHG  1   2  -1
M  END
>  <Similarity_ESP>
0.46565058557634675

>  <Similarity_best>
0.50484238357349409

>  <Similarity_hit>
tasku_input1-agonist

>  <Similarity_shape>
0.54403418157064143

>  <confnum>
2

>  <ID>
phenytoin

>  <PRIMARY_SOM>
14 15 16 17 18 19

>  <Citation>
Komatsu et al.,Drug Metab. Disp.,28,1361,2000

>  <poi>
18:4.0887, 17:4.3518, 14:4.9891, 10:5.4184, 11:5.9408, 8:6.1484, 3:7.4869
, 13:7.7481, 9:7.8527, 5:7.8839, 16:8.4568, 1:8.6516, 12:8.654, 4:9.0017,
6:9.0109, 19:9.204, 15:9.2938, 2:9.415, 7:9.6745

$$$$
```
